# Labour market uncertainty after the irruption of COVID-19

**DOI:** 10.1007/s00181-022-02304-7

**Published:** 2022-09-14

**Authors:** Oscar Claveria, Petar Sorić

**Affiliations:** 1 AQR-IREA, University of Barcelona, Diagonal, 690, 08034 Barcelona, Spain; 2 Faculty of Economics & Business, University of Zagreb, Trg Republike Hrvatske 14, 10000 Zagreb, Croatia

**Keywords:** C14, C32, C82, C83, J01, COVID-19, Labour market uncertainty, Unemployment, Expectations, Consumers, Cointegration

## Abstract

This paper examines the evolution of labour market uncertainty after the irruption of the COVID-19 pandemic in European countries. Since uncertainty is not directly observable, we use two alternative methods to directly approximate it. Both approaches are based on qualitative expectations elicited form the consumer survey conducted by the European Commission. On the one hand, following (Dibiasi and Iselin in Empir Econ 61:2113–2141, 2021), we use the share of consumers unable to formalise expectations about unemployment (Knightian-type uncertainty). On the other, we use the geometric discrepancy indicator proposed by (Claveria in Empirica 48:483–505, 2021) to quantify the proportion of disagreement in business and consumer expectations. We find that both uncertainty measures covary across the 22 European countries analysed. Although we observe differences in the evolution across countries, in most cases the perception of labour market uncertainty peaked before the outbreak of the crisis, plummeted during the first months of the lockdown, and started rising again. When testing for cointegration with the unemployment rate, we find that both indicators exhibit a long-term relationship with unemployment in most countries. The impact of both indicators on unemployment is characterised by considerable asymmetries, showing a more intense reaction to decreases in the level of labour market uncertainty. While this finding may seem counterintuitive at first sight, it somehow reflects the fact that during recessive periods, the level of disagreement in consumer unemployment expectations drops considerably.

## Introduction

The sharp contraction in economic activity triggered by the uncertainty caused by the pandemic has had a major impact on consumer perceptions and the labour market (Hampson et al. 2021; Kim et al. 2021; van der Wielen and Barrios 2021). In spite of the policy measures aimed at supporting workers, the shock on the labour market has been unprecedented, with the unemployment rate exhibiting a sharp increase between February and April 2020. Against this backdrop, analysing consumer unemployment expectations and labour uncertainty seems more timely than ever before.

The notion that the labour market is affected by uncertainty was recently highlighted by the International Labour Organization (2022) when acknowledging that ‘the confluence of various macroeconomic trends is creating uncertainty around whether the drop in working hours, employment and labour force participation is temporary, or whether the pandemic is expediting more structural labour market exits or labour-saving transformations’.

This link between uncertainty and the labour market is well established in theoretical economic models. Starting from the fact that hiring can be regarded as an investment decision for a company (McDonald and Siegel 1986), firms make decisions on when to employ new workers or lay off the current ones. Those decisions are based on comparing workers’ expected return to the associated costs (advertisement, recruitment, training, wages, dismissal costs, etc.). As shown by Ernst and Viegelahn (2014), the minimum positive wedge between return and costs required for firms to acquire new employees depends on the prevailing degree of uncertainty.

Similarly, uncertainty also determines the probability of dismissing existing workers. Within that context, mention should be made of Neumann and Topel (1991), who introduced a theoretical model postulating that unemployment discrepancies among markets are generated by the corresponding differentials of labour demand uncertainty. When testing their model on US state unemployment and employment, the authors found that the covariance structure of sectoral demands for labour, which reflects labour market uncertainty, indeed fed into transitory fluctuations of unemployment.

Uncertainty may also affect economic activity and employment through several transmission mechanisms. Bernanke (1983) was the first to formalise the *real options* mechanism, depicting a situation where firms postpone any type of irreversible decisions with sunk costs—including hiring and dismissal—when faced with high uncertainty. In such circumstances, managers activate the *wait and see* mechanism (Bachman and Bayer 2013), trying to gather any kind of relevant additional information that might decrease uncertainty and potentially boost the expected return of the considered action (see Bloom 2014 for a detailed exposition of this framework). Schaal (2017) noted that the negative impacts of uncertainty could be higher for risk-averse decision makers.

The literature also acknowledges the potential for positive effects of uncertainty for risk seeking agents. For example, the *growth options* framework highlights that high uncertainty may stimulate some agents to intensify investments because uncertainty increases the size of the potential prize (Bloom 2014). This impact should be particularly accentuated for technology- and capital-intensive firms. Likewise, the Oi-Hartman-Abel effect (see Abel 1983; Hartman 1972; Oi 1961) builds upon the concept of hedging, postulating that firms may be risk-loving if they can exploit the possibility to ensure against negative business outcomes. In such specific circumstances, uncertainty may even have positive effects (Bloom 2014).

Within this interplay of the possible negative and positive effects of uncertainty on the labour market, it is extremely hard to disentangle the prevailing aggregate impact, let alone to identify the relevance of each particular transmission mechanism. Schaal (2017) introduced a general equilibrium search-and-matching model for the labour market, allowing for several real-life stylised facts, such as firm dynamics and heterogeneity in firms’ productivity and size. Implementing the model with real US data, Schaal (2017) found mild support for the *real options* channel and the Oi-Hartman-Abel effect, both dominated by what the author called the realised volatility effect. Specifically, the author showed that volatility shocks intensified labour reallocation and led to higher unemployment, and found that uncertainty alone explained as much as 40% of the variation in the US unemployment rate during the global financial crisis.

Despite the existence of a huge and growing literature on the impact of economic uncertainty on activity (Baker et al. 2016; Choi et al. 2021; Carriero et al. 2018, 2022; Ghirelli et al. 2021; Škrabić Perić and Sorić 2017; Zhu et al. 2019), its effect on unemployment has been somehow relegated to the background, in a similar way to unemployment expectations (Abberger 2007; Claveria 2019; Sorić et al. 2019). Some exceptions include the works of Caggiano et al. (2014, 2017a, b), Choi and Loungani (2015), Netšunajev and Glass (2017) and Nodari (2014), which empirically confirm the contribution of economic uncertainty shocks to the volatility of unemployment, especially during recessions. Nevertheless, all these studies focus on the impact of ‘economic uncertainty’, but do not analyse the effect of ‘labour market uncertainty’.

Due to the difficulty of measuring uncertainty, the impact of employment uncertainty shocks on unemployment has largely been overlooked. While some authors have analysed the relationship between oil price shocks and unemployment (Kocaasland 2019), or between exchange rate uncertainty and unemployment (Chang 2011), to our knowledge there is just one previous study that analyses the impact of employment uncertainty on unemployment (Claveria 2021). Therefore, in this study we intend to cover this deficit by measuring and assessing consumer employment uncertainty in European countries. To this end, we use consumer survey expectations of future unemployment as input to calculate employment uncertainty in 22 economies.

Survey-derived measures of expectations dispersion constitute a primary source for eliciting the perceived uncertainty of economic agents, as they present several advantages over alternative methods to proxy such an elusive concept as uncertainty. In this sense, Bloom et al. (2021) have recently used business expectations to measure business’ subjective uncertainty, and at the beginning of the lockdown, Binder (2020) conducted a survey among consumers about their concerns about COVID-19 and their macroeconomic expectations.

On the one hand, the forward-looking nature of consumer expectations makes them particularly useful for computing survey-derived measures of expectations dispersion (Binding and Dibiasi 2017; Clements and Galvão 2017). Unlike economic uncertainty measures based on the volatility in equity markets (Basu and Bundick 2017; Bekaert et al. 2013; Caggiano et al. 2014), or on the conditional volatility of the unforecastable components of economic variables (Jurado et al. 2015; Meinen and Roehe 2017; Rossi and Sekhposyan 2015), survey-based proxies allow an ex-ante analysis of the effects of uncertainty on the economy.

On the other hand, there is recent evidence that different dimensions of uncertainty have different effects on the economy (Henzel and Rengel 2017). In this regard, Claveria (2021) has shown the suitability of addressing the analysis of each type of uncertainty independently, as the aggregation of expectations both from different agents (businesses and consumers) and from various economic variables to approximate economic uncertainty may end up causing the effect of the different dimensions of uncertainty on activity to be compensated.

As a result, in this study we exclusively use consumers’ unemployment expectations elicited from the consumer tendency survey conducted by the European Commission and compute two different measures of employment uncertainty. We use two alternative approaches recently proposed in the literature. First, we use an indicator that directly measures Knightian uncertainty. The indicator is informed by the measure proposed by Dibiasi and Iselin (2021), although it is not based on the knowledge of past developments since we do not have access to the micro data. As suggested by the authors, uncertainty in the sense of Knight (1921) is defined by a situation in which agents are no longer able to form expectations about the future. Therefore, through the measurement of the proportion of respondents who explicitly state that they ‘do not know’ what the expected direction of their unemployment expectations is, we compute a first indicator of consumer labour uncertainty.

Second, we compute a disagreement measure of consumer unemployment expectations. With this aim we apply the geometric approach proposed by Claveria (2021). This method allows for calculating a dimensionless metric that gives the proportion of discrepancy among survey respondents, where zero corresponds to the point of minimum consumers’ disagreement, and one indicates that the answers are equidistributed among the different response categories.

The prospective nature of survey expectations, together with the availability of information regarding consumers’ unemployment expectations, has allowed us to focus on this overlooked aspect in such a critical moment as the present, a year after the irruption of the COVID-19 pandemic. In the study we examine the evolution of consumers’ perceived uncertainty about employment and its relation to that of the unemployment rate. To this aim, we make use of nonlinear econometric techniques to test for cointegration between labour uncertainty and unemployment. This approach allows us to test for the existence of a long-term relationship between both variables in the main European economies. To our knowledge, this is the first study to compare these two recent metrics of consumer employment uncertainty and to analyse their relationship with the unemployment rate.

The remainder of the paper is structured as follows. The next section presents the data and analyses the two proxies of employment uncertainty. Section 3 presents the methodology used to evaluate the long-term relationship between both metrics and the unemployment rate. The empirical results are presented in Sect. 4. Finally, Sect. 5 concludes the study.

## Data

The European Commission conducts monthly business and consumer tendency surveys in which respondents are asked whether they expect different economic variables to rise, fall or remain unchanged. In the present study, we focus on consumers’ qualitative expectations about future unemployment. Specifically, we use the raw data from 2005.M1 to 2021.M12 for 22 European economies, the EA and EU. We used year-on-year growth rates of Gross Domestic Product (GDP) and harmonised seasonally adjusted unemployment rates (UN) provided by the Organisation for Economic Co-operation and Development (OECD) (https://stats.oecd.org/index.aspx?queryid=36324). The different countries have been denoted as follows: Belgium (BE), Czech Republic (CZ), Denmark (DK), Germany (DE), Estonia (EE), Greece (EL), Spain (ES), France (FR), Italy (IT), Latvia (LV), Lithuania (LT), Luxemburg (LU), Hungary (HU), the Netherlands (NL), Austria (AT), Poland (PL), Portugal (PT), Slovenia (SI), Slovakia (SK), Finland (FI), Sweden (SE), the United Kingdom (UK), the Euro Area (EA) and the European Union (EU). For the UK there is only available information up until 2020.M12.

In consumer tendency surveys, respondents are asked about their expectations about how the level of unemployment will change in their country over the next 12 months. Consumers are faced with six options: $PP_{t}$ measures the percentage of respondents reporting a sharp increase in the variable, $P_{t}$ a slight increase, $E_{t}$ no change, $M_{t}$ a slight fall, $MM_{t}$ a sharp fall and, $N_{t}$ don’t know. Survey results are usually published as balances, which can be regarded as a diffusion index consisting of the subtraction between the aggregate percentages of response corresponding to the extreme categories (see Pinto et al. 2020 for a comprehensive analysis of diffusion indexes). For consumers, the balance is computed as follows:

1 $$B_{t} = \left( {PP_{t} + {\raise0.7ex\hbox{$1$} \!\mathord{\left/ {\vphantom {1 2}}\right.\kern-\nulldelimiterspace} \!\lower0.7ex\hbox{$2$}}P_{t} } \right) - \left( {{\raise0.7ex\hbox{$1$} \!\mathord{\left/ {\vphantom {1 2}}\right.\kern-\nulldelimiterspace} \!\lower0.7ex\hbox{$2$}}M_{t} + MM_{t} } \right)$$

Seasonally adjusted balances are published each month by the Commission, but the series corresponding to each response category are only available in raw form, that is, the aggregate percentage of respondents in each category. Since both metrics of labour market uncertainty are computed from raw data, which are not seasonally adjusted, we have used the X-13ARIMA-SEATS filter in order to extract the periodicities that are closest to those observed in seasonally adjusted unemployment rates. In Appendix 2 we compared the results with those resulting from using a Butterworth filter. See Claveria et al. (2021) for a justification of this type of filter for the analysis of business and consumer survey data.

Many studies on economic uncertainty rely on quantitative macroeconomic expectations made by professional forecasters to compute dispersion-based proxies (Dovern 2015; Lahiri and Sheng 2010; Oinonen and Paloviita 2017). However, consumer tendency surveys provide qualitative measures of agents’ expectations, and therefore the measures of disagreement among survey respondents mainly use the dispersion of balances as a proxy for uncertainty (Bachmann et al. 2013; Girardi and Reuter 2017; Mokinski et al. 2015).

This idea was first suggested by Theil (1955), who proposed using a disconformity coefficient. In their seminal paper, Bachmann et al. (2013) applied an indicator of disagreement based on the square root of the variance of the balance, which in the case of the consumers would be computed as follows:

2 $$DISP_{t} = \sqrt {\left( {PP_{t} + {\raise0.7ex\hbox{$1$} \!\mathord{\left/ {\vphantom {1 2}}\right.\kern-\nulldelimiterspace} \!\lower0.7ex\hbox{$2$}}P_{t} } \right) + \left( {{\raise0.7ex\hbox{$1$} \!\mathord{\left/ {\vphantom {1 2}}\right.\kern-\nulldelimiterspace} \!\lower0.7ex\hbox{$2$}}M_{t} + MM_{t} } \right) - (B_{t} )^{2} }$$

The fact that expression (2) does not include the share of neutral responses ($E_{t}$) causes the level of disagreement to be overestimated, as shown by means of a simulation experiment in Claveria et al. (2019). Therefore, in this study, we use the disagreement metric proposed by Claveria (2021), which incorporates the information coming from all the reply options. Based on the fact that the sum of the shares adds up to one, and that the vector encompassing all shares of responses can be projected onto a simplex, the author proposed using the barycentre system to geometrically derive the ratio of agreement among respondents as the distance of the vector to the centre of the simplex divided by the distance from the centre to the nearest vertex. For simplicity, we group all ‘positive’ and ‘negative’ answers, adding *P* and *PP* and *M* and *MM* to reduce the number of response categories. By equidistributing *N* between the three groups (increase, decrease and no change), we neutralise the effect of introducing that share together with the ‘no-change’ category and mixing different information. In Appendix 1 we compared the results with those resulting from adding *E* and *N* in the no-change category. This way, an indicator of disagreement for a given period in time can be formalised as:

3 $$D_{t} = 1 - \left[ {\frac{{\sqrt {\left( {PP_{t} + P_{t} + {\raise0.7ex\hbox{${N_{t} }$} \!\mathord{\left/ {\vphantom {{N_{t} } 3}}\right.\kern-\nulldelimiterspace} \!\lower0.7ex\hbox{$3$}} - {\raise0.7ex\hbox{$1$} \!\mathord{\left/ {\vphantom {1 3}}\right.\kern-\nulldelimiterspace} \!\lower0.7ex\hbox{$3$}}} \right)^{2} + \left( {E_{t} + {\raise0.7ex\hbox{${N_{t} }$} \!\mathord{\left/ {\vphantom {{N_{t} } 3}}\right.\kern-\nulldelimiterspace} \!\lower0.7ex\hbox{$3$}} - {\raise0.7ex\hbox{$1$} \!\mathord{\left/ {\vphantom {1 3}}\right.\kern-\nulldelimiterspace} \!\lower0.7ex\hbox{$3$}}} \right)^{2} + \left( {MM_{t} + M_{t} + {\raise0.7ex\hbox{${N_{t} }$} \!\mathord{\left/ {\vphantom {{N_{t} } 3}}\right.\kern-\nulldelimiterspace} \!\lower0.7ex\hbox{$3$}} - {\raise0.7ex\hbox{$1$} \!\mathord{\left/ {\vphantom {1 3}}\right.\kern-\nulldelimiterspace} \!\lower0.7ex\hbox{$3$}}} \right)^{2} } }}{{\sqrt {{\raise0.7ex\hbox{$2$} \!\mathord{\left/ {\vphantom {2 3}}\right.\kern-\nulldelimiterspace} \!\lower0.7ex\hbox{$3$}}} }}} \right]$$

One of the main advantages of this metric is that is bounded between zero and one, and therefore directly interpretable: 0 is the point of minimum disagreement among consumers, when one category draws all the answers, and 1 the point of maximum disagreement in which the answers are equidistributed among the three response categories.

When comparing the evolution of the geometric measure of disagreement (3) to that of the standard deviation of the balance (2) in several European countries, Claveria (2021) obtained a high positive correlation between both measures of disagreement, and found that the main difference between both measures was mainly in their average level and dispersion, with *DISP* higher and more volatile than *D*.

As commented in the Introduction, Dibiasi and Iselin (2021) proposed using the share of respondents that, when surveyed, explicitly responded that they did not know the expected direction of their expectations with the aim of obtaining a direct measure of Knightian uncertainty. Hence, in this study we use the share of consumers that respond that they do not know the expected direction of their unemployment expectations (*N*), which captures the proportion of consumers that are not able to formalise expectations about the future unemployment level. See Dibiasi and Iselin (2021) for a comparison of (2) to Theil’s disconformity coefficient and a thorough analysis of firms’ direct perception of investment uncertainty. For the sake of comparability, we normalise *N*.

In Table 1, we present the summary statistics of the proxies of employment uncertainty: geometric disagreement in consumer unemployment expectations (*D*) and the normalised proportion of consumers who explicitly manifest that they do not know how the level of unemployment will change in their country over the next 12 months (*N*). On the one hand, results in Table 1 show that overall, the proportion of disagreement tends to be high among consumers, well above 50% in all countries except Greece, Italy, and Portugal. Notwithstanding this, the dispersion of *N* is higher than that of *D* in many countries.

**Table 1:** Descriptive analysis (2005.01–2021.12)

Country	*N*		*D*		Unemployment	
	mean	SD	mean	SD	mean	SD
Belgium	0.378	0.270	0.593	0.291	7.393	1.090
Czechia	0.342	0.297	0.556	0.251	5.123	2.011
Denmark	0.297	0.233	0.665	0.179	5.837	1.386
Germany	0.444	0.226	0.737	0.244	5.854	2.334
Estonia	0.338	0.237	0.657	0.227	7.881	3.429
Greece	0.245	0.191	0.446	0.288	17.174	6.681
Spain	0.277	0.315	0.634	0.257	17.076	5.613
France	0.396	0.249	0.574	0.297	9.078	0.919
Italy	0.284	0.250	0.476	0.244	9.603	2.089
Latvia	0.491	0.224	0.670	0.190	10.538	4.144
Lithuania	0.354	0.203	0.758	0.246	9.377	3.830
Luxemburg	0.387	0.195	0.609	0.260	5.383	0.751
Hungary	0.540	0.188	0.665	0.218	7.040	2.628
Netherlands	0.419	0.237	0.602	0.288	5.158	1.228
Austria	0.564	0.242	0.588	0.243	5.258	0.706
Poland	0.408	0.209	0.686	0.162	8.093	3.887
Portugal	0.417	0.284	0.478	0.338	10.669	3.318
Slovenia	0.294	0.289	0.530	0.282	6.775	1.937
Slovakia	0.383	0.261	0.679	0.252	11.019	3.271
Finland	0.437	0.241	0.655	0.212	7.909	0.953
Sweden	0.279	0.240	0.692	0.193	7.459	0.935
UK	0.472	0.344	0.550	0.266	5.776	1.468
EA	0.363	0.229	0.647	0.256	9.380	1.533
EU	0.425	0.233	0.649	0.250	8.879	1.516

SD denotes standard deviation. *D* refers to disagreement regarding consumers’ ‘unemployment expectations over the next 12 months’ and *N* refers to the normalised share of consumers’ that choose the ‘I do not know’ category in the consumer survey. UK denotes the United Kingdom, EA the Euro Area, and EU the European Union

**Fig. 1:**
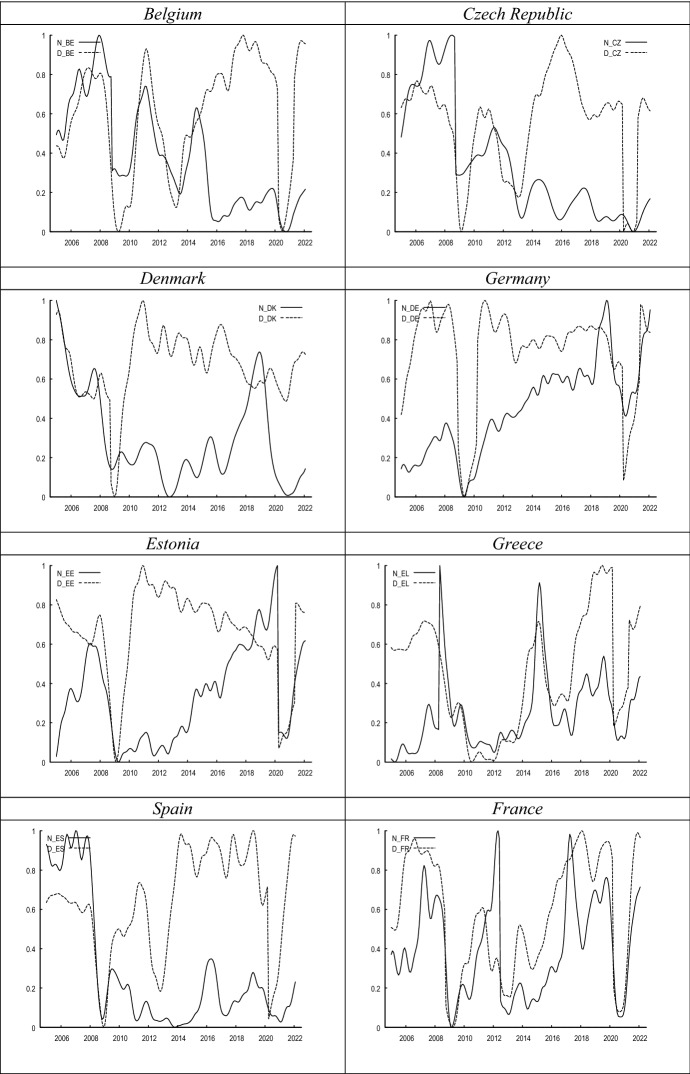

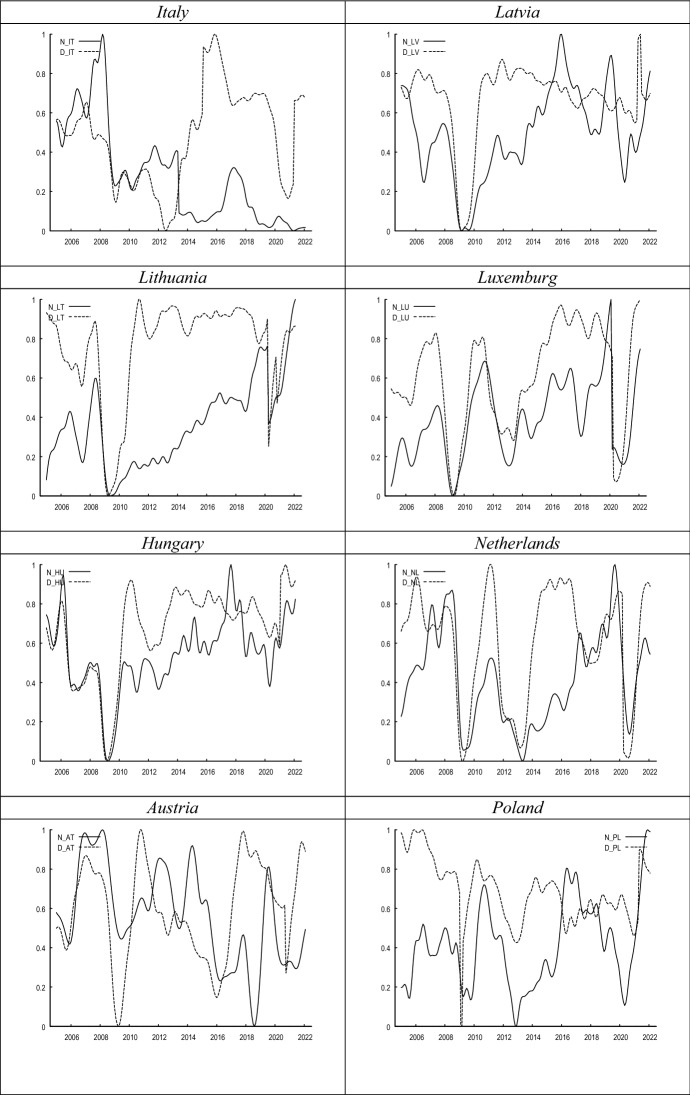

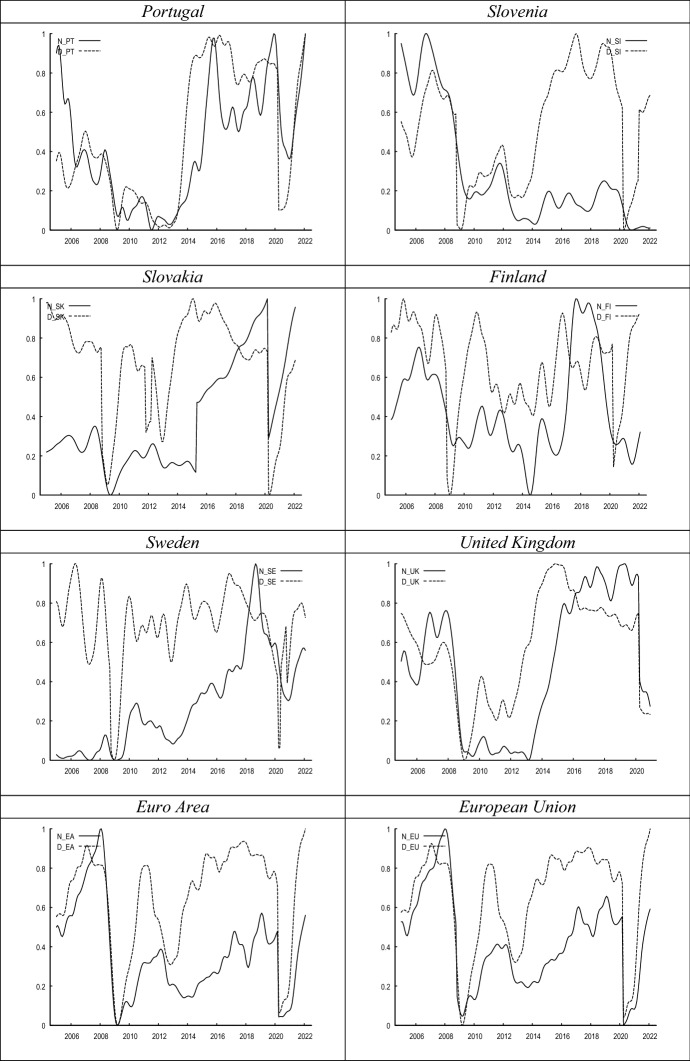
Evolution of *N* versus *D*. *Notes* *N*_country refers to the normalised share of consumers’ that choose the ‘I do not know’ category in the consumer survey regarding ‘unemployment expectations over the next 12 months’; *D*_country represents the evolution of disagreement amongst consumers’ unemployment expectations. Both series have been smoothed with the X-13 filter

**Fig. 2:**
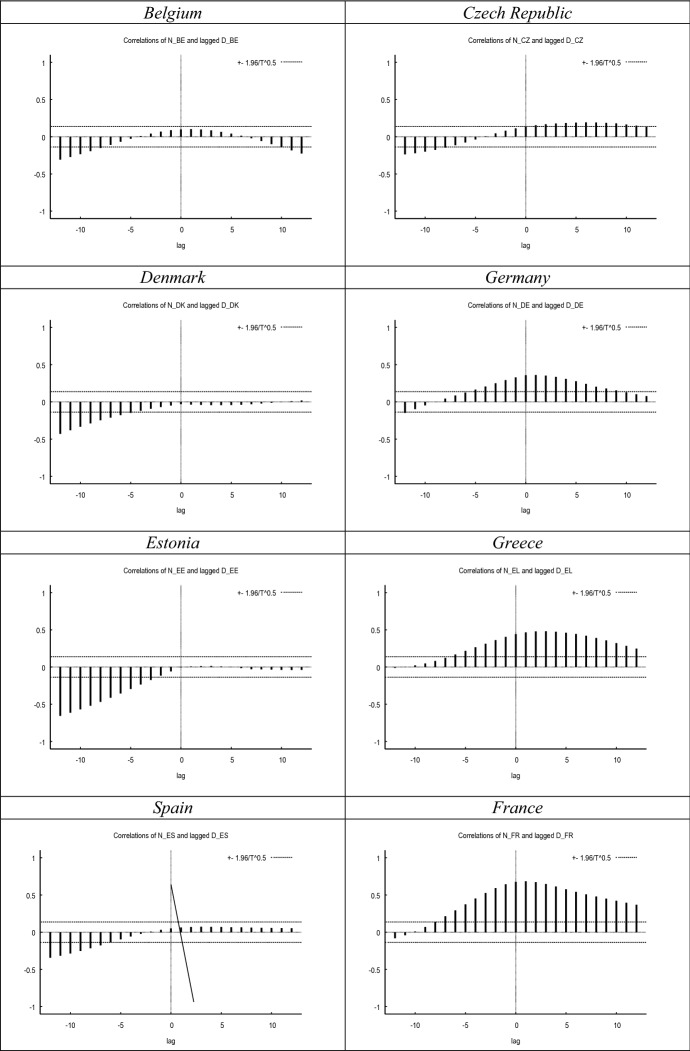

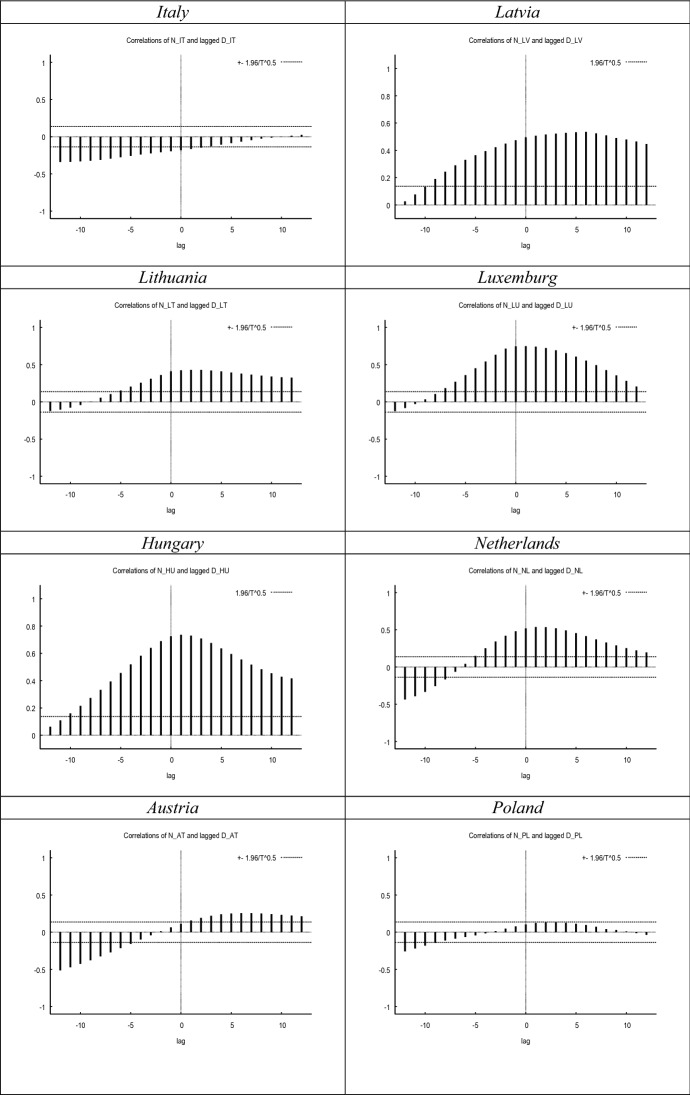

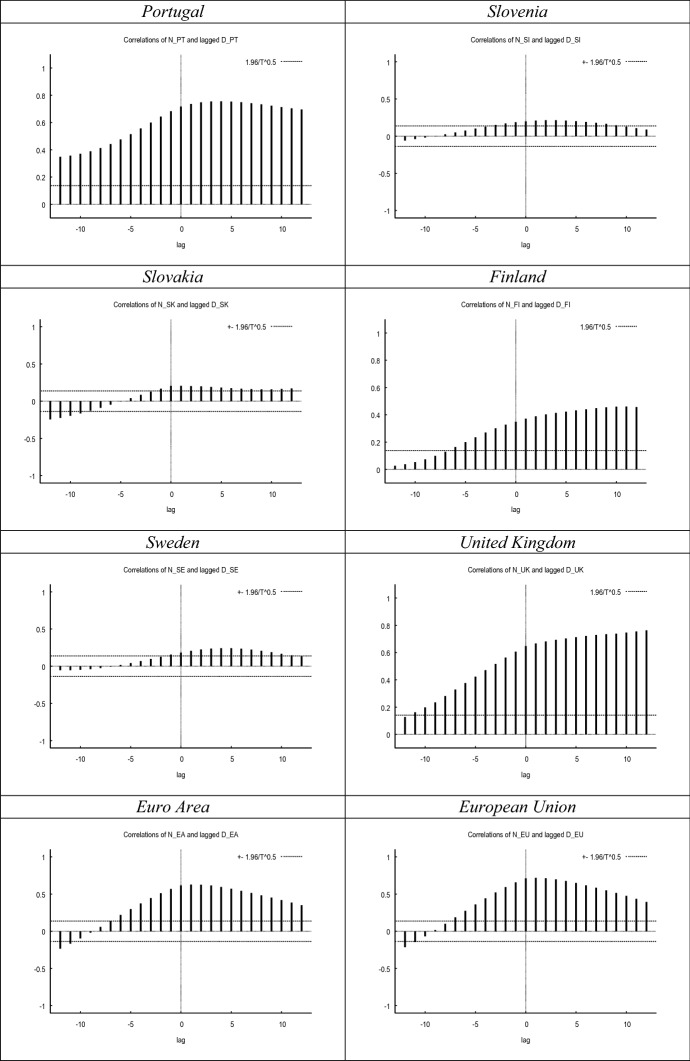
Cross-correlograms–*N* versus lagged *D*. *Notes* *N* and *D* have been smoothed with the X-13 filter. Maximum correlation and corresponding period between brackets. All maximum correlations significant at the 0.01 level (2-tailed). Cross-correlations for the UK up until 2020.12

Figure 1 compares the evolution of both proxies of labour market uncertainty. While in most countries both metrics seem to covary during the sample period, in other countries the correlation between both metrics seems to be weak. This notion is further confirmed in Fig. 2, where we show the cross-correlograms between both measures. The graphs in Fig. 1 show a high concordance between both indicators at the inflection points, corresponding to periods of extreme uncertainty such as the 2008 crisis or the current one.

Finally, we run the Kwiatkowski–Phillips–Schmidt–Shin (KPSS) test on both proxies of labour market uncertainty and unemployment for each country. Results are presented in new Table 2. The KPSS test for stationarity (Kwiatkowski et al. 1992) tests the null hypothesis that the time series is stationary around a deterministic trend against the alternative of a unit root. Cases in which the null hypothesis of stationarity cannot be rejected at the 5% significance level are marked in bold. While in most cases the null hypothesis is rejected, we obtain mixed evidence for all three variables *N*, *D* and unemployment.

**Table 2:** Test for stationarity–KPSS test statistics

Country	*N*		*D*		Unemployment	
	With trend	No trend	With trend	No trend	With trend	No trend
Belgium	**0.089**	2.796	0.158	0.472	0.524	1.847
Czechia	0.318	3.174	0.260	**0.250**	0.510	2.907
Denmark	0.486	0.969	0.274	**0.298**	0.753	0.819
Germany	0.181	3.182	**0.123**	**0.125**	0.751	3.786
Estonia	0.407	1.256	0.368	**0.425**	0.424	0.868
Greece	**0.108**	0.581	0.542	0.783	0.862	1.952
Spain	0.715	2.143	0.250	0.760	0.966	1.260
France	0.153	**0.202**	0.347	**0.392**	0.765	0.795
Italy	0.212	2.829	0.368	0.781	0.789	2.243
Latvia	0.324	1.073	0.194	**0.271**	0.569	0.938
Lithuania	0.532	2.284	0.267	0.752	0.602	0.730
Luxemburg	**0.094**	0.967	0.168	0.600	0.277	2.529
Hungary	0.285	1.087	0.201	1.654	0.788	2.527
Netherlands	0.413	**0.433**	0.152	**0.147**	0.595	0.592
Austria	**0.142**	1.579	0.176	**0.236**	**0.146**	0.708
Poland	**0.142**	0.540	0.317	1.338	0.280	2.987
Portugal	0.563	1.437	0.339	1.697	0.926	1.100
Slovenia	0.706	2.693	0.326	0.668	0.842	0.847
Slovakia	0.531	2.690	0.295	**0.299**	0.555	2.654
Finland	0.322	**0.315**	0.230	**0.363**	0.349	**0.392**
Sweden	0.199	3.114	0.163	**0.185**	0.254	**0.275**
UK	0.575	1.360	0.394	1.070	0.781	1.647
EA	0.348	0.829	0.203	**0.324**	0.873	0.896
EU	0.319	0.604	0.196	**0.259**	0.793	1.134

Estimation period 2005.01–2021.12. Kwiatkowski–Phillips–Schmidt–Shin (KPSS) test for stationarity (Kwiatkowski et al. 1992). Critical values (i) with trend: 0.120 (10%), 0.148 (5%), 0.217 (1%); (ii) with no trend: 0.348 (10%), 0.462 (5%), 0.739 (1%). Null hypothesis: time series is stationary around a deterministic trend (i.e. the process is trend-stationary), against the alternative of a unit root. Cases in which the null hypothesis of stationarity cannot be rejected at the 5% significance level are marked in bold

## Methodology

In this section, we present the methodology used to test for the existence of a long-term relationship between unemployment uncertainty and the unemployment rate, henceforth denoted as UN. Our estimation strategy is largely conditioned by the fact that the assessed dataset is consisted of a mixture of stationary and integrated time series, i.e. *I*(0) and *I*(1). This prevented us from framing the study within a standard Johansen cointegration or vector autoregression (VAR) analysis, and led us to use an autoregressive distributed lag (ARDL) model.

As explained in the Introduction, based on both an economic theory and an empirical perspective, despite the scarce existing literature to date, uncertainty is found to be a major driver of labour market developments. Nevertheless, although the short-run consequences of uncertainty are mostly well described, its long-run effects are still less clear. In this sense, although the related literature mostly finds negative short-term uncertainty effects on economic activity, Bloom (2014) stated that the *real options* channel, through decreased consumption and increased savings, might even trigger a rise in long-term investments, which should consequentially lead to higher growth.

Additionally, Bloom (2014) showed that the Oi-Hartman-Abel effect should be particularly strong in the medium to long run. Schaal (2017) particularly emphasised the long-term nature of the relationship between uncertainty and the labour market, since employment contracts usually refer to longer periods of time and involve considerable long-run costs. Thus, from these theoretical postulates, one would expect that uncertainty has significant long-run effects on economic activity, particularly regarding the labour market. As a result, we have opted for an ARDL modelling approach.

The proposed ARDL methodology has some noteworthy benefits. On the one hand, it allows for a combination of *I*(0) and *I*(1) variables (Pesaran et al. 2001); on the other hand, it also preserves valuable degrees of freedom by allowing for different lag orders for each variable at hand. Besides, the ARDL model is robust to bi-directional feedback effects between dependent variable and regressors, conditioned to a correct specification of the lag order so that regressors become weakly exogenous (see Chudik et al. 2016; Mohaddes et al. 2022).

Additionally, the ARDL approach allows for augmenting the model in a nonlinear fashion. Namely, previous studies of economic uncertainty have unequivocally demonstrated the asymmetric impact of economic uncertainty on aggregate economic activity (Jones and Enders 2016; Caggiano et al. 2017a, [Bibr CR19][Bibr CR20]; Forni et al. 2021; Jackson et al. 2020), finding a stronger effect for increases in uncertainty than for decreases.

Therefore, in order to capture these potential asymmetries, we use the nonlinear ARDL (NARDL) framework of Shin et al. (2014):

4 $$\Delta UN_{t} = a_{0} + \rho UN_{t - 1} + \theta^{ + } X_{t - 1}^{ + } + \theta^{ - } X_{t - 1}^{ - } + \mathop \sum \limits_{j = 1}^{p - 1} a_{j} \Delta UN_{t - j} + \mathop \sum \limits_{j = 0}^{{q^{ + } - 1}} \pi_{1,j}^{ + } \Delta X_{t - j}^{ + } \; + \;\mathop \sum \limits_{j = 0}^{{q^{ - } - 1}} \pi_{1,j}^{ - } \Delta X_{t - j}^{ - } \; + \;e_{t} ,$$

where *X* $$=\left\{\begin{array}{c}D\\ N\end{array}\right.$$, ${X}_{t}^{+}=\sum_{j=1}^{t}\mathrm{max}\left(\Delta {x}_{j},0\right)$ and${X}_{t}^{-}=\sum_{j=1}^{t}\mathrm{min}\left(\Delta {x}_{j},0\right)$. Model (4) was estimated for each country in the sample and for each of the two uncertainty proxies (*D* and *N*). The optimal lag order of the NARDL model (*p*, $q^{ + }$ and $q^{ - }$) was determined via the general-to-specific approach (Greenwood-Nimmo and Shin 2013; Shin et al. 2014). Model (4) was estimated in a stepwise fashion, starting from *p* = $q^{ + }$ = $q^{ - }$ = 6 and then iteratively dropping all insignificant regressors with a 5% significance stopping rule. This type of modelling strategy was suggested by Greenwood-Nimmo and Shin (2013) and Greenwood-Nimmo et al. (2013), because including insignificant lags to the NARDL specification may likely induce spurious results and add noise to the model. It should be noted that this kind of NARDL specification corrects for potential weak endogeneity of explanatory variables (Shin et al. 2014), so it is also robust to feedback effects between the dependent variable and regressors. The null hypothesis of no cointegration (${{\varvec{H}}}_{0}:$ $\rho = \theta^{ + } = \theta^{ - } = 0$) is tested by a standard Wald test.

A novelty of NARDL in comparison to linear ARDL is the necessity to test for long-run symmetry (${{\varvec{H}}}_{0}:$ $\theta^{ + } = \theta^{ - } )$ and short-run symmetry ($\mathop \sum \nolimits_{j = 0}^{{q^{ + } - 1}} \pi_{1,j}^{ + } = \mathop \sum \nolimits_{j = 0}^{{q^{ - } - 1}} \pi_{1,j}^{ - }$), again by means of a Wald test. Greenwood-Nimmo et al. (2013) suggested to test for both types of (a)symmetries (long- and short-run), and then to re-estimate Eq. (4) if only one type of asymmetry (or none) is found. This should prevent the researcher from obtaining biased results due to model misspecifications. If the null hypothesis of long-run symmetry cannot be rejected, we therefore re-estimate Eq. (4) as:

5 $$\Delta UN_{t} = a_{0} + \rho UN_{t - 1} + \theta X_{t - 1} + + \mathop \sum \limits_{j = 1}^{p - 1} a_{j} \Delta UN_{t - j} \; + \;\mathop \sum \limits_{j = 0}^{{q^{ + } - 1}} \pi_{1,j}^{ + } \Delta X_{t - j}^{ + } \; + \;\mathop \sum \limits_{j = 0}^{{q^{ - } - 1}} \pi_{1,j}^{ - } \Delta X_{t - j}^{ - } \; + \;e_{t} ,$$

Similarly, in case the short-run symmetry cannot be rejected, we re-estimate the model as:

6 $$\begin{aligned} \Delta UN_{t} = & a_{0} + \rho UN_{t - 1} + \theta^{ + } X_{t - 1}^{ + } + \theta^{ - } X_{t - 1}^{ - } \\ & + \mathop \sum \limits_{j = 1}^{p - 1} a_{j} \Delta UN_{t - j} + \mathop \sum \limits_{j = 0}^{q - 1} \pi_{1,j} \Delta X_{t - j} + e_{t} , \\ \end{aligned}$$

Finally, if both types of symmetries cannot be rejected, we estimate the purely linear ARDL model:

7 $$\Delta UN_{t} = a_{0} + \rho UN_{t - 1} + \theta X_{t - 1} + \mathop \sum \limits_{j = 1}^{p - 1} a_{j} \Delta UN_{t - j} + \mathop \sum \limits_{j = 0}^{q - 1} \pi_{1,j} \Delta X_{t - j} + e_{t} ,$$

Upon estimating a separate NARDL model for each country, two diagnostic tests are carried out for each NARDL model: a Ljung–Box test for autocorrelation of 12th order, and an Engle’s Autoregressive Conditional Heteroscedasticity (ARCH) test of 12th order. Whenever the residuals turned out to be characterised by autocorrelation or heteroscedasticity at the 5% significance level, the Newey–West autocorrelation- and heteroscedasticity-consistent (HAC) estimator was used.

As the final step of our empirical strategy, conditional on the presence of significant asymmetries (short-run, long-run, or both), we estimate responses of unemployment to positive and negative unit changes in consumer employment uncertainty (${X}_{t}^{+}$ and ${X}_{t}^{-}$). With the aim of empirically testing whether unemployment indeed reacts asymmetrically to consumer uncertainty measures, we compute the dynamic multipliers:

8 $$m_{h}^{ + } = \mathop \sum \limits_{j = 0}^{h} \frac{{\partial UN_{t + j} }}{{\partial X_{t}^{ + } }}\;{\text{and}}\;m_{h}^{ - } \mathop \sum \limits_{j = 0}^{h} \frac{{\partial UN_{t + j} }}{{\partial X_{t}^{ - } }},\;h = 0,\; 1,\; 2,\; \ldots$$

Given that the proposed measures of labour market uncertainty might contain valuable information for explaining other aspects of aggregate economic activity, in Appendix 3 the NARDL analysis presented through Eqs. (4), (5), (6), (7) and (8) was replicated using GDP year-on-year growth rates as the dependent variable instead of UN.

## Results

In this section, we present the empirical results of the NARDL cointegration analysis. Table 3 summarises the results for the impact of *D* on the unemployment rate, while Table 4 presents analogous results for the relationship between *N* and unemployment. In all models, we have controlled for economic growth. We used the Chow and Lin (1971) interpolation technique to extract monthly data from quarterly GDP. Instead of presenting the obtained parameters for each individual lag of each of the variables included in the models, we summarised the main findings by presenting only the nature of the final model specification, i.e. whether there are significant asymmetries in the model, the *F* statistics associated to the cointegration tests, and the long-run consumer employment uncertainty parameters.

**Table 3:** NARDL cointegration analysis results–Effect of *D* on unemployment

Country	Type of asymmetry	Cointegration test *F* value	$\theta^{ + }$	$\theta^{ - }$
Austria	SR^HAC^	13.29**	0.1908	–
Belgium	none^HAC^	19.26 **	− 0.2595**	–
Czechia	SR	7.79**	− 0.1902*	–
Denmark	SR^HAC^	28.28**	− 0.1583	–
Estonia	SR^HAC^	19.63**	− 0.2445	–
Finland	SR^HAC^	6.44**	− 0.4989**	–
France	(SR, LR)^HAC^	55.83**	− 0.1742**	− 0.4659**
Germany	SR	11.51**	− 0.0875	–
Greece	SR	17.21**	− 0.4989**	–
Hungary	SR^HAC^	8.46**	− 0.6044**	–
Italy	SR^HAC^	25.35**	− 0.2775**	–
Latvia	(SR, LR)^HAC^	4.03	1.2399**	− 0.1085
Lithuania	SR, LR	6.46**	− 0.4509*	0.0133
Luxembourg	SR	2.86	− 0.1161**	–
Netherlands	none^HAC^	11.44**	− 0.0351	–
Poland	(SR, LR)^HAC^	8.63**	− 0.0062	0.0445
Portugal	SR^HAC^	11.22**	− 0.2565**	–
Slovakia	SR, LR	15.56**	− 0.1863**	− 0.1120*
Slovenia	SR, LR	9.64**	− 0.4669**	− 0.0817
Spain	none^HAC^	0.94	− 0.0843	–
Sweden	none^HAC^	7.57**	− 0.3227*	–
UK	SR^HAC^	3.90	− 0.1193	–
EA	none^HAC^	1.70	− 0.0837	–
EU	SR	2.24	− 0.1258*	–

**Significance at the 0.01 level, * at the 0.05 level. ^HAC^ denotes a model estimated using the Newey–West standard error correction due to autocorrelation and/or heteroscedasticity issues. ‘–’ denotes no negative effect, i.e. a unique (symmetric) long-run coefficient is estimated. Full set of results is available upon request

**Table 4:** NARDL cointegration analysis results–Effect of *N* on unemployment

Country	Type of asymmetry	Cointegration test *F* value	$\theta^{ + }$	$\theta^{ - }$
Austria	SR^HAC^	4.28	− 0.0455	–
Belgium	SR	8.49**	0.0325	–
Czechia	LR	16.83**	− 0.9627**	− 0.3542**
Denmark	SR^HAC^	15.27**	− 0.0524	− 0.0520**
Estonia	SR^HAC^	17.16**	0.0115	–
Finland	SR^HAC^	11.11**	− 0.6722**	–
France	SR^HAC^	2.02	− 0.2355**	–
Germany	LR^HAC^	16.49**	− 0.1055*	− 0.0122**
Greece	SR^HAC^	15.43**	− 0.0602	–
Hungary	none^HAC^	4.82	− 0.5163**	–
Italy	SR^HAC^	13.72**	0.0218	–
Latvia	(SR, LR)^HAC^	1.82	− 0.1800	− 0.1042
Lithuania	none	13.72**	− 0.2378*	–
Luxembourg	SR	0.54	− 0.0018	–
Netherlands	(SR, LR)^HAC^	9.75**	− 0.4115**	− 0.8128**
Poland	(SR, LR)^HAC^	9.97**	− 0.1379	0.0730
Portugal	none^HAC^	8.23**	− 0.2653**	–
Slovakia	none^HAC^	14.15**	− 0.2820**	–
Slovenia	LR^HAC^	8.43**	− 0.5042**	− 0.2304**
Spain	none^HAC^	2.24	0.0049	–
Sweden	SR^HAC^	12.57**	0.0681	–
UK	LR	7.16**	− 0.2732**	− 0.3474**
EA	SR, LR	8.40**	0.2738**	0.0412
EU	SR	1.33	0.0338	–

**Significance at the 0.01 level, * at the 0.05 level. ^HAC^ denotes a model estimated using the Newey–West standard error correction due to autocorrelation and/or heteroscedasticity issues. ‘–’ denotes no negative effect, i.e. a unique (symmetric) long-run coefficient is estimated. Full set of results is available upon request

The results in Table 3 suggest that employment uncertainty approximated via disagreement (*D*) is cointegrated with unemployment in all countries except Latvia, Luxembourg and Spain, as well as the UK, EA, and EU. The estimated long-run coefficients are negative in the majority of countries with a significant long-term relationship, implying that a rise in disagreement is associated with a decrease in the unemployment rate. While this finding may seem counterintuitive at first, there may be a plausible explanation related to both the choice of uncertainty proxy, and the fact that the obtained results may be also reflecting that consumers’ expectations become more uniform in relation to employment during periods of severe recession such as the current one, aligning around a pessimistic perspective. See Alonso et al. (2017) and Dube and Black (2010) for an evaluation of the differences in consumer perception before and after national traumatic events.

Disagreement in forecasting between economic agents’ is often regarded as a proxy for economic uncertainty (Bachmann et al. 2013). Bloom (2014) established counter-cyclicality as one of the fundamental stylised facts of economic uncertainty. In this sense, we want to note that the obtained results could be somehow suggesting that highly heterogeneous survey responses regarding unemployment expectations may not always indicate high labour market uncertainty. This notion is also in line with recent evidence indicating that forecast disagreement and news-based indicators of uncertainty capture inherently different phenomena (Glas 2020; Krüger and Nolte 2016; Rich and Tracy 2021; Sorić and Lolić 2017). Although Bachmann et al. (2013) found an expected countercyclical impact of their disagreement indicator on industrial production, follow-up studies often detected a discrepancy between disagreement and other uncertainty proxies, so some authors insist on segregating these two concepts (Jurado et al. 2015; Krüger and Nolte 2016). Regardless of that, the computed measure of disagreement did indeed include valuable information for the long-run state of unemployment, and this result was very robust across different countries.

**Fig. 3:**
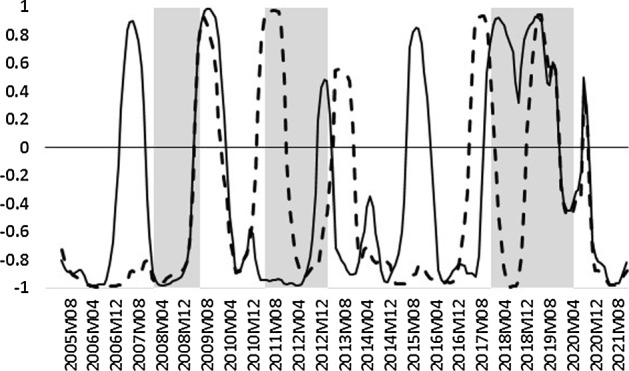
Rolling-window correlation of uncertainty measures and unemployment for the EA. *Notes* Solid line represents the correlation between *DIS* and the unemployment rate in the EA. Dashed line represents the correlation between *D* and the unemployment rate in the EA. Shaded areas correspond to recessions (*source*: Federal Reserve Economic Data)

In this sense, to shed some light on the potential reasons for a negative relationship between the assessed uncertainty measures and unemployment, we calculated the rolling correlation between the two stated series. The obtained results for the EA are presented in Fig. 3, along with the shaded areas corresponding to recessions.

In Fig. 3, it can be seen that the correlation between uncertainty and unemployment plummets into negative territory at the beginning of recessions. It seems that once the economic outlook reaches its trough, uncertainty levels also drop (see Fig. 1), since consumers’ expectations of the immediate future look so pessimistic that there is hardly any uncertainty regarding the direction of unemployment. This pattern is also observed quite regularly during recessions, such as, for example, in the 2008 crisis, as well as in the European sovereign debt crisis, and the recent pandemic-induced turbulences.

As the relationship between uncertainty and unemployment is obviously dependent on the business cycle, this type of behaviour brings our attention to the possible asymmetries in the observed relationship. It may be the case that unemployment generally reacts differently to increases and decreases in uncertainty. Tables 3 and 4 reveal several significant asymmetries—both in the short and the long run—in the impact of both assessed uncertainty proxies on unemployment. With the aim of further exploring this question, we calculated the dynamic multipliers (see Eq. (8)).

**Fig. 4:**
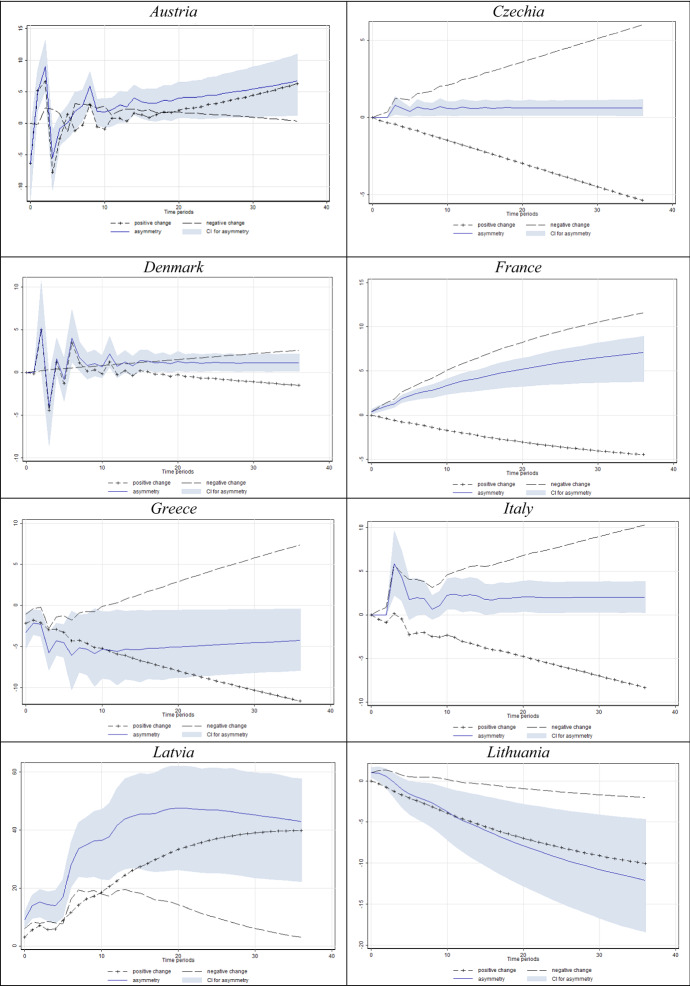

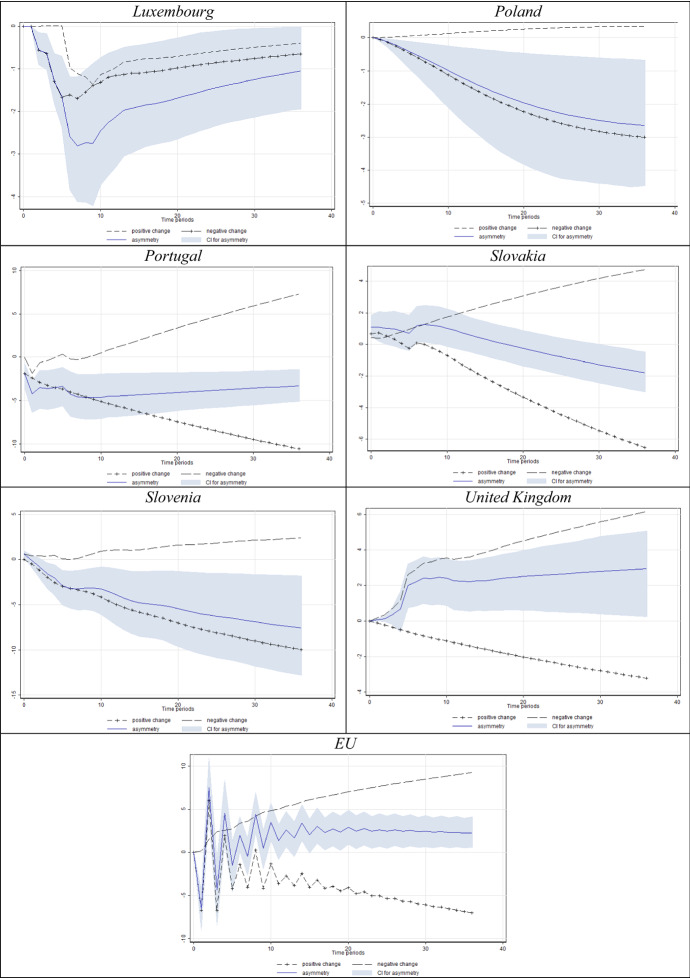
Estimated dynamic multipliers–Effect of *D* on unemployment. *Note* Shaded area corresponds to the 95% confidence interval (CI) for asymmetry (difference between positive and negative effect)

**Fig. 5:**
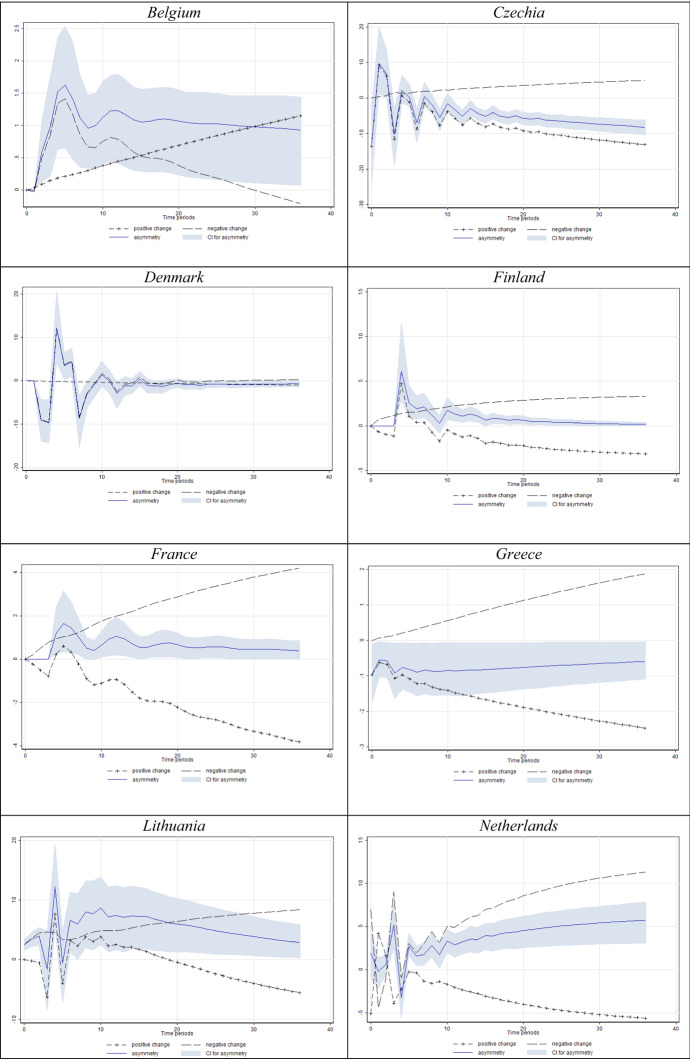

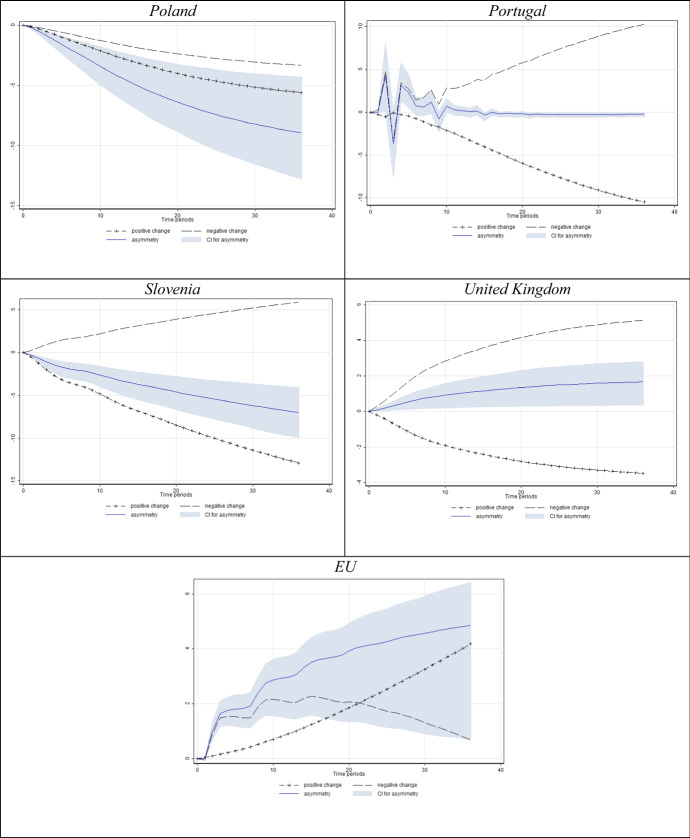
Estimated dynamic multipliers–Effect of *N* on unemployment. *Note* Shaded area corresponds to the 95% confidence interval (CI) for asymmetry (difference between positive and negative effect)

In Figs. 4 and 5 we present the results for the models in which we found a significant short- and/or long-run asymmetry. For clarity, we only present significant short- and/or long-run asymmetry according to the Wald test, corroborated by the dynamic multipliers whose 95% confidence interval does not comprise the value of zero (implying significance at the 5% level).

The graphical presentations provided in Fig. 4 reveal that unemployment mostly rises in response to decreasing labour market uncertainty, and drops in response to increasing uncertainty. In this sense, the obtained results are very similar to those presented in Tables 3 and 4. Additionally, we want to note that we proxy employment uncertainty using consumers’ disagreement regarding their unemployment expectations. Our data suggest that consumers generate the most homogeneous expectations during extreme events such as recessions. Due to such behaviour, a fall in forecasting disagreement corresponds to an increase in actual unemployment.

When it comes to the dynamic multipliers of unemployment in response to *N* (Fig. 5), the results are somewhat similar. Again, for a vast majority of countries with significant asymmetries (95% asymmetry confidence interval not including zero), unemployment seems to react more intensively to decreases in Knightian uncertainty. The share of consumers unable to formalise expectations about unemployment considerably falls in economic downturns (see Fig. 1). This finding drives the negative sign of uncertainty parameters in most specifications in Table 3, again with a significant cointegrating equation in most specifications.

The obtained results showed that both the disagreement indicator and Knightian uncertainty were cointegrated with unemployment in most of the countries, whereas the former regressor offers more abundant evidence. As the real economy undergoes the expected downfall, consumers seem almost unified in the belief that the situation will worsen, hence generating low levels of disagreement, while at the same time unemployment actually rises. This combination of effects ultimately yields a negative relationship between consumer labour market uncertainty and unemployment during recessions, which obviously conditions the overall negative long-run relationship between the two variables.

To further validate the results obtained, two additional robustness checks are carried out in Appendices 3 and 4. To analyse whether the proposed measures of labour market uncertainty possess valuable information for explaining other aspects of aggregate economic activity, in Appendix 3 we estimated the NARDL models using GDP year-on-year growth rates as the dependent variable. To further assess the robustness of our results, in Appendix 4 we estimated a VAR model with a recursive identification scheme. The VAR model, without error correcting dynamics, provides information on the short-run relationship among the variables. The results presented in Appendices 3 and 4 corroborate the significant relationship with unemployment and the positive effect of both uncertainty proxies on economic growth.

The hereby established dependence of our results on the economic cycle is somewhat in line with the results obtained by Forni et al. (2021). Just as we have found that extreme negative events drive the negative relationship between labour market uncertainty and unemployment, Forni et al. (2021) showed that the relationship between uncertainty and economic activity is particularly moderated by the left tail of the growth forecast distribution. Moreover, the fact that consumers’ disagreement regarding their unemployment expectations and Knightian labour uncertainty show a positive association with economic activity could also be linked to herding behaviour. In this sense, Rülke et al. (2016) found evidence that professional macroeconomic forecasters tend to exhibit herding behaviour in times of economic turbulence, producing very similar forecasts and lowering the overall heterogeneity of responses when faced with the threat of a recession.

Despite the lack of studies on this issue, research that links economic uncertainty with activity, and more specifically with unemployment, yields similar results. Therefore, our estimates are in line with previous studies. Chang (2011) analysed the relationship between exchange rate uncertainty and unemployment for South Korea and Taiwan, obtaining a long-run equilibrium relationship. Hayford (2000) used the variance of the unemployment forecasts of the Livingston survey to proxy unemployment uncertainty and analysed its effect on economic activity, finding that it was Granger-caused by inflation. Our findings are also in line with theoretical macroeconomic models, indicating the interconnections between different types of uncertainty (Henzel and Rengel 2017; Sánchez 2012). In a recent article, Claveria (2021) also used unemployment expectations from consumers surveys to proxy unemployment uncertainty. The author found that shocks in unemployment uncertainty were found to lead to a decrease in unemployment rates, but that they were of smaller magnitude than those of economic uncertainty or of inflation uncertainty.

Theoretical literature recognises that integrating forecast disagreement into macroeconomic models helps the general understanding of the economic cycle. This has been shown for producer prices (Woodford 2003), financial markets (Scheinkman and Xiong 2003), and GDP (Dovern et al. 2012). However, the literature is completely silent on the importance of forecast disagreement for unemployment. Our paper provides an initial contribution in that context, finding that the proposed uncertainty proxies add to the understanding of unemployment and GDP patterns.

The relevance of the proposed proxies of labour market uncertainty does not stem purely from the empirical results or their statistical significance. Our findings also highlight the relevance of theoretical concepts such as information rigidity and imperfect information, as opposed to the new classical notion of full information and rational expectations. Namely, a growing body of literature is dealing with concepts such as sticky information (Mankiw and Reis 2002) and noisy information (Sims 2003; Woodford 2003). The former framework accentuates the cost of updating agents’ information sets, while the latter one explicates rational inattention through agents’ limited ability to filter valuable information from noisy signals. Both information frictions could be influencing the labour market uncertainty proxies proposed in the present study, since they could considerably increase the heterogeneity of agents' unemployment expectations, as well as disabling agents to formalise their unemployment expectations. The effective contrast of these hypotheses would be based on experimental evidence, along the lines of the work of Khaw et al. (2017), this being a prospective future direction of research.

The examination of the asymmetries between consumer labour market uncertainty and unemployment has so far been an unexplored issue. However, previous studies that analyse the link between unemployment and other types of uncertainty also detected the existence of asymmetries. Ahmed et al. (2020) examined how different economic uncertainty measures affected the unemployment level in the United States across all states of the business cycle, employing linear and nonlinear causality-in-quantiles tests. The authors found that the uncertainty-unemployment level relationship was nonlinear and that shocks to economic uncertainty had a negative impact on unemployment outflow, concluding that the effect of economic uncertainty was asymmetric depending on the states. Similarly, Kocaasland (2019) also found that unemployment rates reacted asymmetrically to positive and negative shocks on oil price uncertainty.

## Conclusion

COVID-19 has had an unprecedented effect in unemployment and consumer expectations. In this context, the analysis of the perception of labour market uncertainty by consumers is of particular interest. Since employment uncertainty is unobservable, there is a gap in the literature on its analysis. This omission has led us to focus on the approximation of labour market uncertainty and the analysis of its effect on unemployment. To this end, we have made exclusive use of consumers’ unemployment expectations elicited from tendency surveys, in which consumers are asked about the expected direction of different economic variables. Using the different shares of responses (increase, decrease, no change) as the sole input, we computed a disagreement metric and compared it to a direct indicator of Knightian labour market uncertainty, which is computed as the share of consumers who are not able to formalise expectations about future unemployment.

By isolating the ‘employment’ dimension of uncertainty and focusing exclusively on consumers’ expectations, we were able to compute two proxies of employment uncertainty to evaluate their effect on unemployment on 22 European countries, the EA and the EU. The potential existence of asymmetries between both variables and the different integration orders of the time series across countries have led us to use nonlinear cointegration analysis. The use of a NARDL framework in this context contrasts with previous research focused on the effects of economic uncertainty on economic activity, which was mainly conducted within a VAR framework.

Therefore, the contributions of this study focus on four different aspects. These include, first, the measurement of labour market uncertainty, and second, the comparison between two recent metrics of uncertainty based on consumer expectations. Third, these elements incorporate the evaluation of the effects of uncertainty on unemployment through nonlinear econometric techniques in order to capture the potential existence of asymmetries between both variables, and finally, the estimation of the responses of unemployment to positive and negative unit changes in uncertainty by computing the dynamic multipliers.

Regarding the first point, it should be noted that both measures of uncertainty are conceptually different. While the metric of discrepancy is based on the geometric derivation of the degree of disagreement between consumers when formulating their expectations, the second proxy elicits the so-called Knightian uncertainty, that is, the proportion of consumers unable to form expectations about employment. Notwithstanding this, when comparing both indicators, we found that they are highly correlated in most countries.

Regarding the evaluation of the existence of a long-term relationship between employment uncertainty and unemployment, we used nonlinear cointegration analysis. The results showed that both the disagreement indicator and Knightian uncertainty are cointegrated with unemployment in most of the countries. As the real economy undergoes the expected downfall, consumers seem almost unified in the belief that the situation will worsen, hence generating low levels of disagreement, while at the same time unemployment actually rises. This combination of effects ultimately yields a negative relationship between consumer employment uncertainty and unemployment during recessions, which obviously conditions the overall negative long-run relationship between the two variables.

Since both indicators of uncertainty showed considerable asymmetries in their effect on unemployment, we finally estimated the responses of the latter to positive and negative unit changes in consumer uncertainty. The estimated dynamic multipliers showed that for both proxies the unemployment rate reacted more intensively to a decrease in uncertainty among consumers. Although this might seem unusual at first glance, our analysis revealed that employment uncertainty measured via consumer disagreement substantially decreased during recessions. This evidence indicates that consumers’ perception becomes more homogeneous when expecting an increase in unemployment. This finding may also suggest a herd behaviour in which consumers’ unemployment expectations tend to align around a pessimistic perception during recessions.

The present study sheds some light on the measurement of labour market uncertainty and its relation to unemployment. This has so far been an overlooked aspect. In such a critical moment as the present, after the sudden outbreak of COVID-19, when European economies are implementing damage contention measures aimed at supporting workers and at mitigating the unprecedented shock on economic activity, this analysis is particularly pertinent. However, the study is not without limitations. Above all, we want to note that the findings of this research may be conditioned by several biases derived from the exogenous measurement of employment uncertainty.

While the main aim of the research was to compare both proxies of uncertainty and their effects on unemployment, an important issue left for further research is the application of alternative approaches to approximate labour market uncertainty, such as the estimation of the unforecastable components of the unemployment rate. The analysis could also be extended to other tendency surveys, such as industry, service or retail trade ones. Given the availability of consumer survey data by strata according to age, income, education, gender, and occupation, in future works we aim to replicate the analysis for different socio-demographic groups of consumers.

## Figures and Tables

**Fig. 6:**
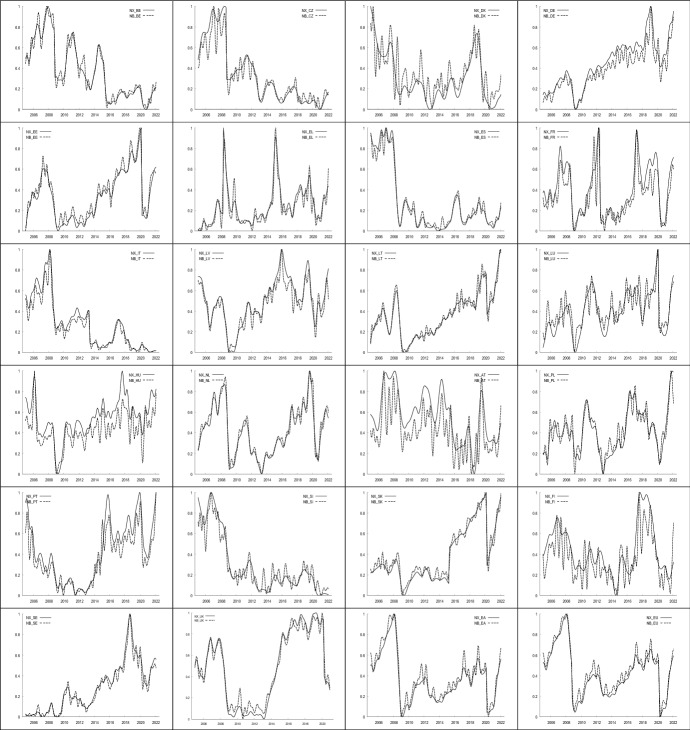
Evolution of *NB* (*N* Butterworth-filtered) versus *NX* (*N* X-13 filtered)

**Fig. 7:**
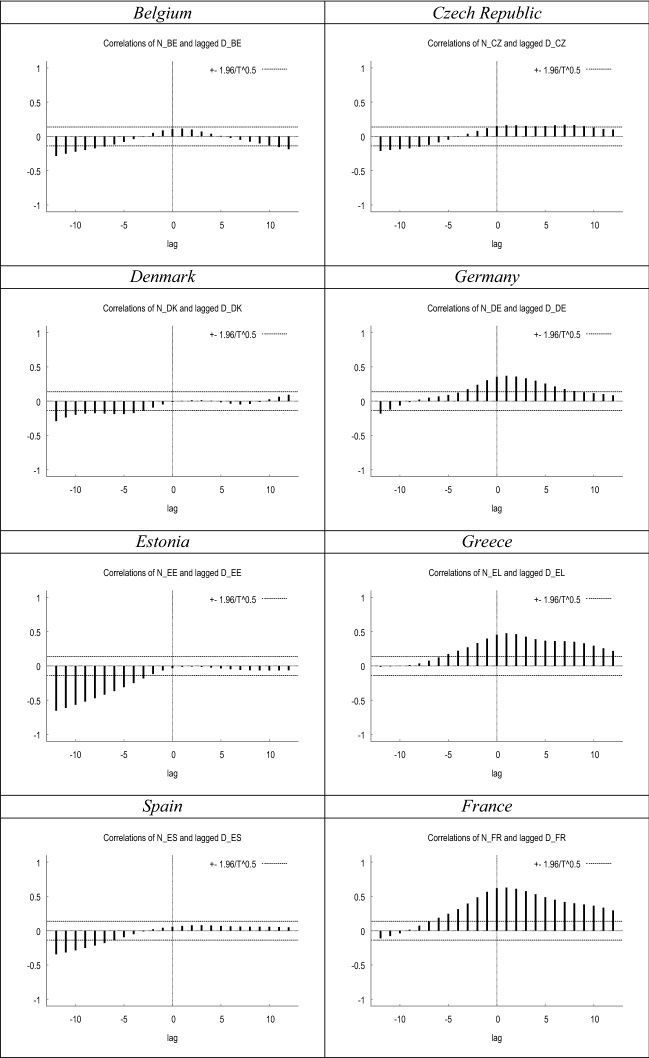

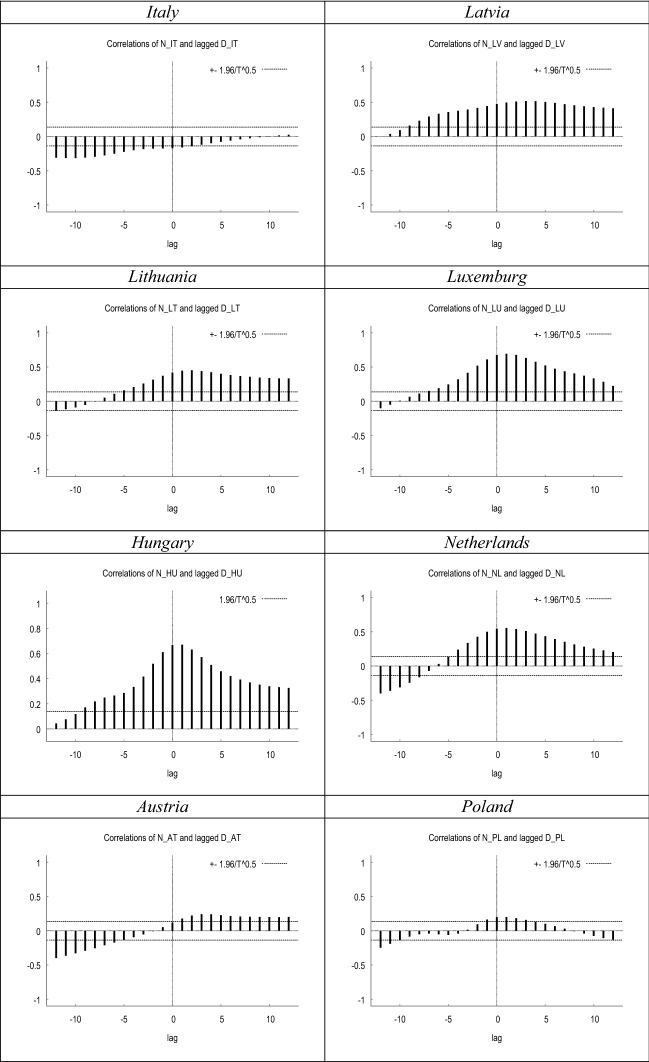

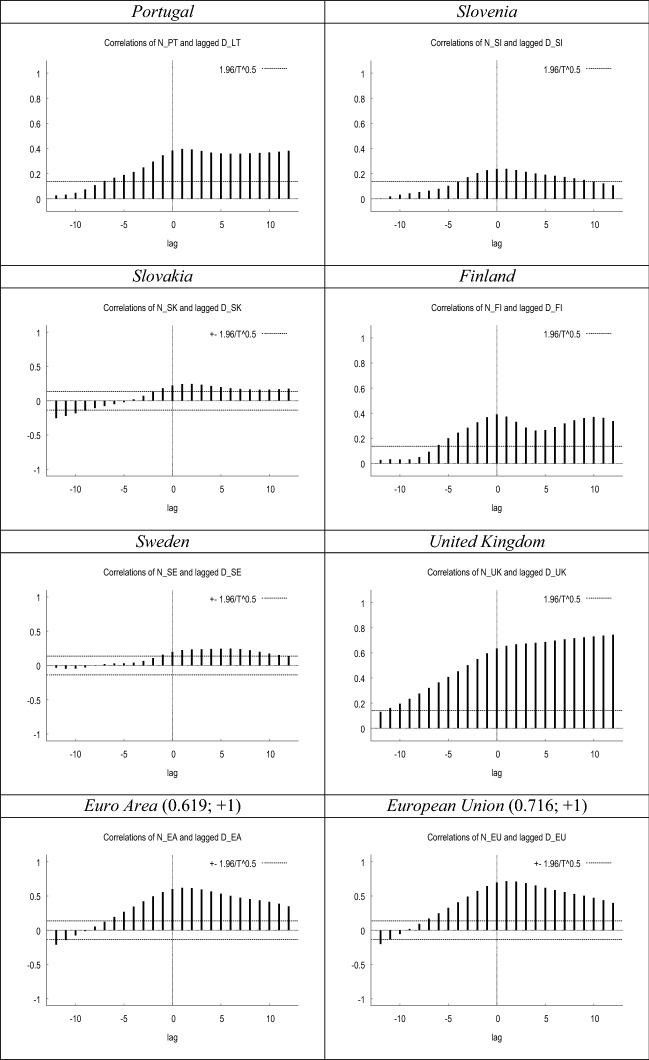
Cross-correlograms–*NB* versus lagged *DB*. *Notes* *NB* and *DB* have been smoothed with the Butterworth filter. Maximum correlation and corresponding period between brackets. All maximum correlations significant at the 0.01 level (2-tailed). Cross-correlations for the UK up until 2020.12

**Fig. 8:**
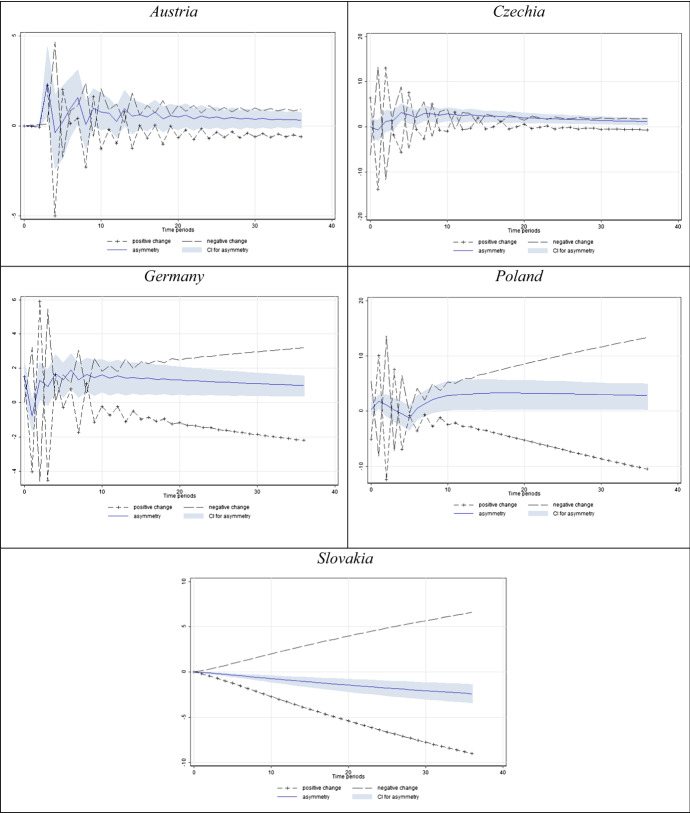
Estimated dynamic multipliers–Effect of *DB* on unemployment. *Note* Shaded area corresponds to the 95% confidence interval (CI) for asymmetry (difference between positive and negative effect)

**Fig. 9:**
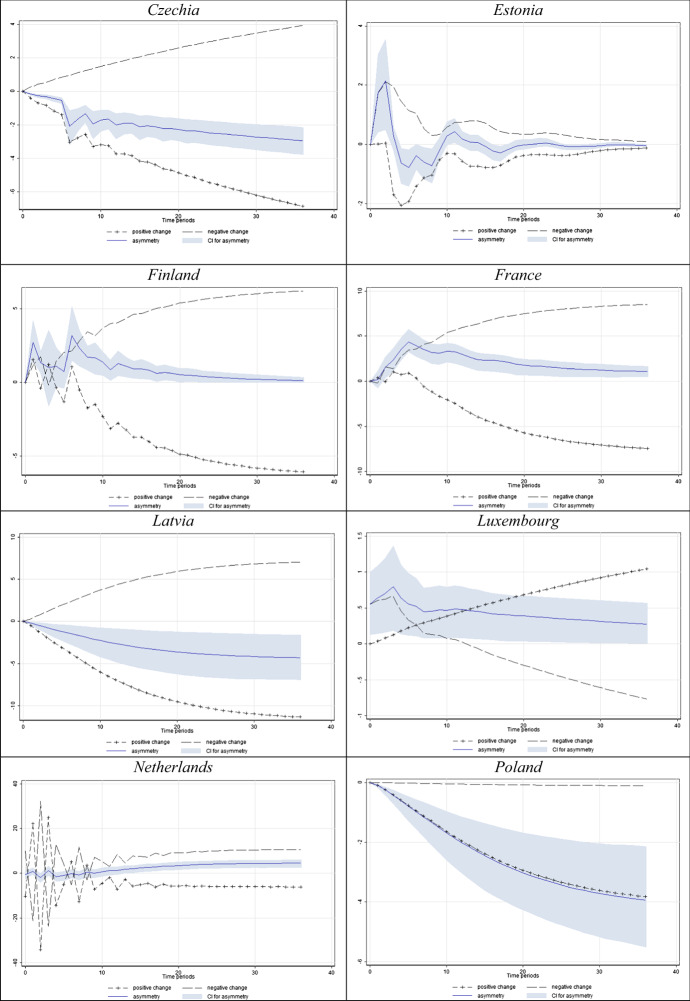

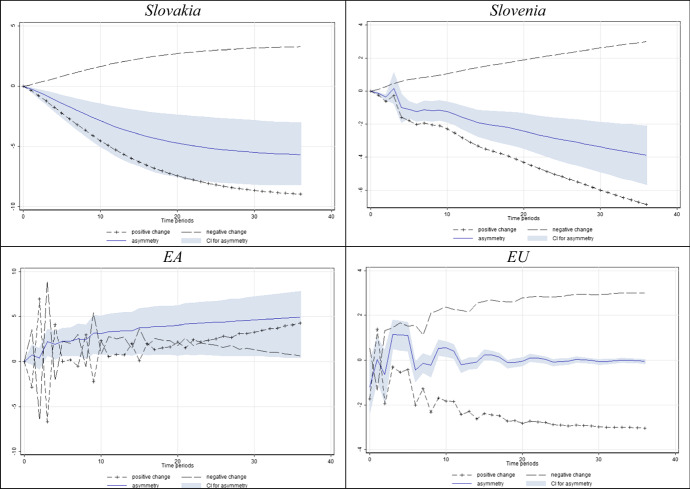
Estimated dynamic multipliers–Effect of *NB* on unemployment. *Note* Shaded area corresponds to the 95% confidence interval (CI) for asymmetry (difference between positive and negative effect)

**Fig. 10:**
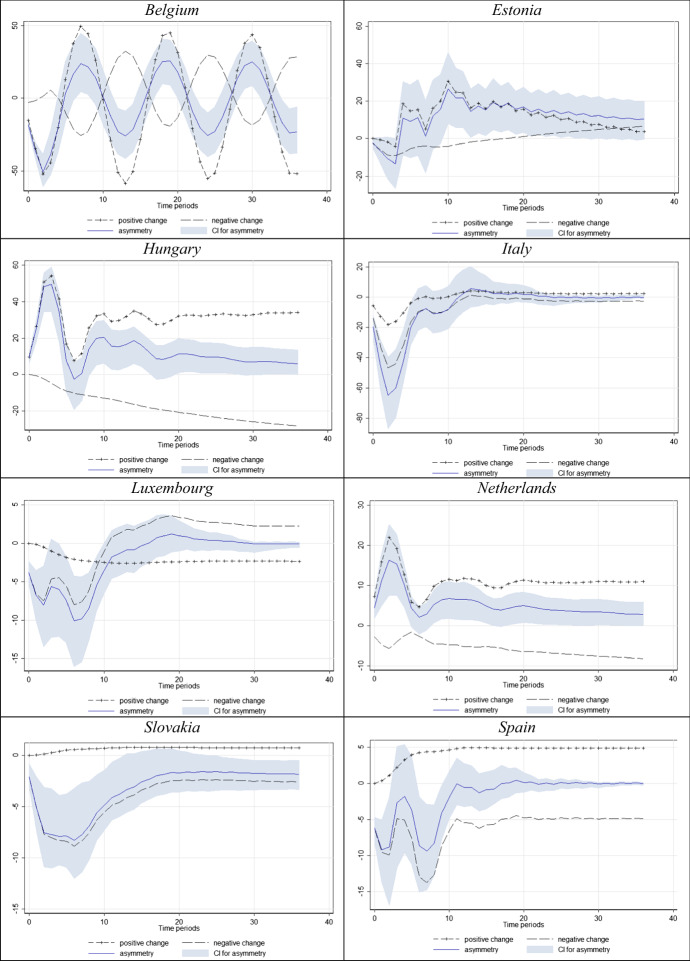

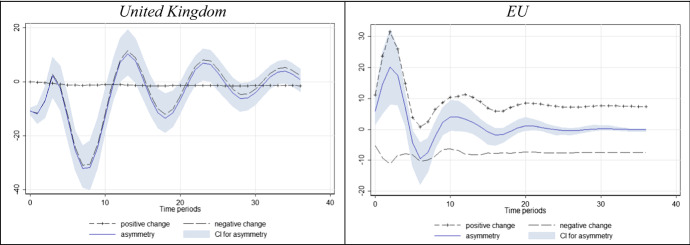
Estimated dynamic multipliers–Effect of *DX* on GDP. *Note* Shaded area corresponds to the 95% confidence interval (CI) for asymmetry (difference between positive and negative effect)

**Fig. 11:**
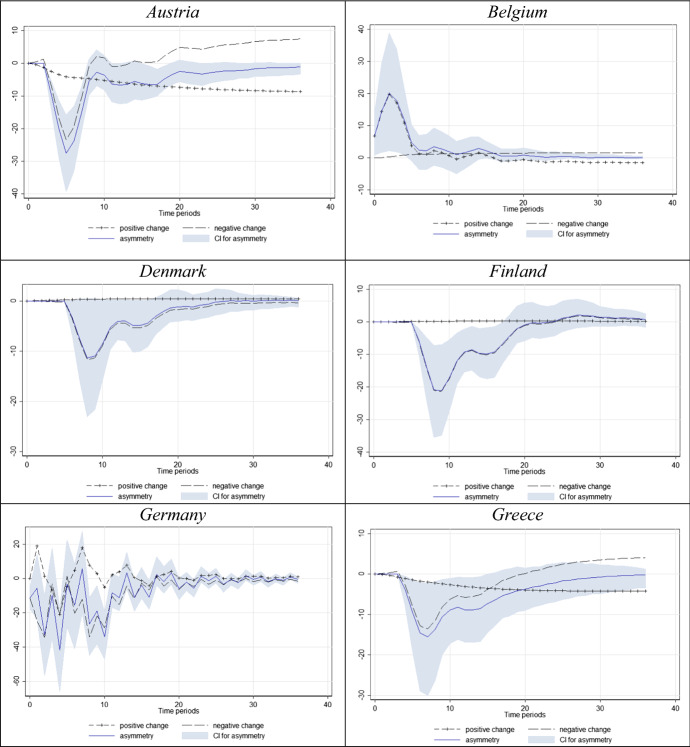

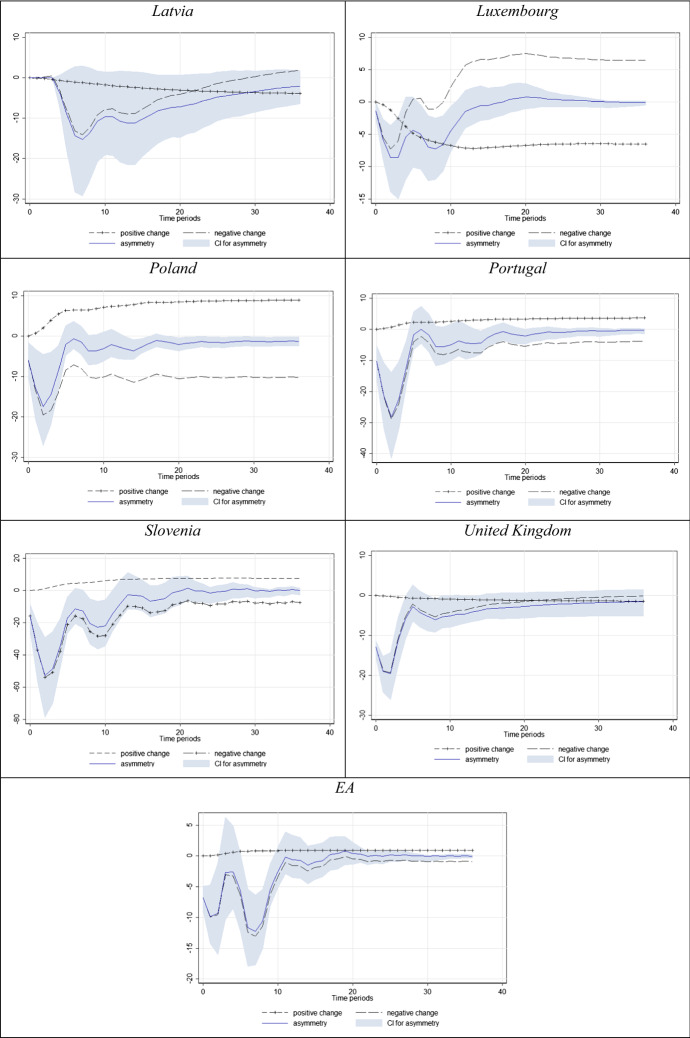
Estimated dynamic multipliers–Effect of *NX* on GDP. *Note* Shaded area corresponds to the 95% confidence interval (CI) for asymmetry (difference between positive and negative effect)

**Fig. 12:**
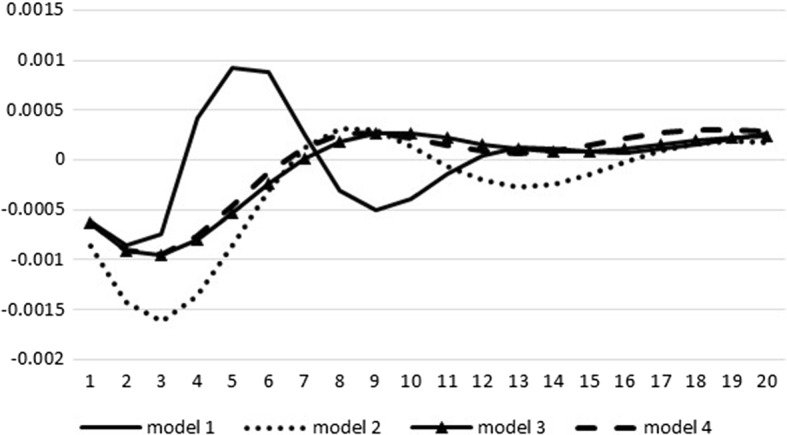
IRF of labour cost to a shock in labour market uncertainty

**Fig. 13:**
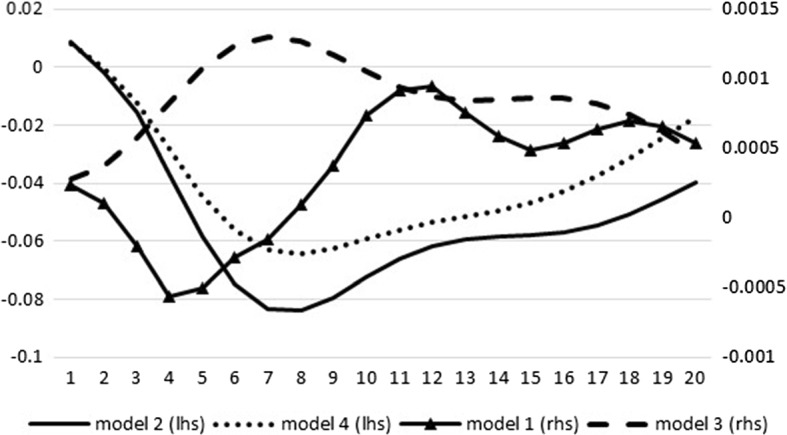
IRF of unemployment to a shock in labour market uncertainty

**Fig. 14:**
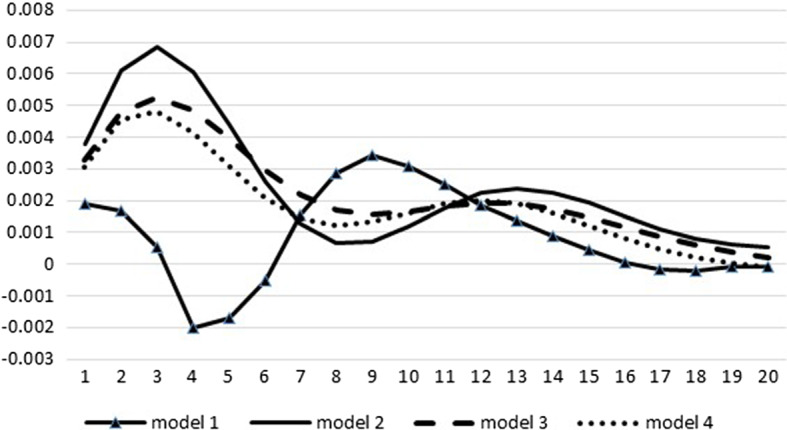
IRF of GDP to a shock in labour market uncertainty

## Data Availability

The datasets used and/or analysed during the current study are: The *Joint Harmonised EU Consumer Survey* conducted by the European Commission, which can be freely downloaded at: https://ec.europa.eu/info/business-economy-euro/indicators-statistics/economic-databases/business-and-consumer-surveys_en. Unemployment rate provided by the OECD at: https://stats.oecd.org/index.aspx?queryid=36324. Gross Domestic Product (GDP) from the Quarterly National Accounts (OECD): https://stats.oecd.org/Index.aspx?DataSetCode=SNA_TABLE1.

## Appendix 1

In order to test the robustness of the obtained results, we first compute the disagreement indicator in a different way. While the proposed measure of consumer disagreement (*D*) in Eq. (3) is calculated so that the share of non-responses is equalised between the different categories rather than aggregated with the no-change proportion (*E*), we now compute the geometric measure of disagreement adding *P* and *PP*, *M* and *MM*, and *E* and *N.* This way, the alternative indicator of disagreement for a given time period can be formalised as:

A1 $$DIS_{t} = 1 - \left[ {\frac{{\sqrt {\left( {PP_{t} + P_{t} - {\raise0.7ex\hbox{$1$} \!\mathord{\left/ {\vphantom {1 3}}\right.\kern-\nulldelimiterspace} \!\lower0.7ex\hbox{$3$}}} \right)^{2} + \left( {N_{t} + E_{t} - {\raise0.7ex\hbox{$1$} \!\mathord{\left/ {\vphantom {1 3}}\right.\kern-\nulldelimiterspace} \!\lower0.7ex\hbox{$3$}}} \right)^{2} + \left( {MM_{t} + M_{t} - {\raise0.7ex\hbox{$1$} \!\mathord{\left/ {\vphantom {1 3}}\right.\kern-\nulldelimiterspace} \!\lower0.7ex\hbox{$3$}}} \right)^{2} } }}{{\sqrt {{\raise0.7ex\hbox{$2$} \!\mathord{\left/ {\vphantom {2 3}}\right.\kern-\nulldelimiterspace} \!\lower0.7ex\hbox{$3$}}} }}} \right]$$

We assess the difference between the two metrics during the sample period. Table

**Table 5:** Difference between *D* and *DIS*

Country	mean	SD
Belgium	0.002	0.003
Czechia	0.004	0.005
Denmark	0.000	0.001
Germany	0.002	0.001
Estonia	0.010	0.007
Greece	0.002	0.005
Spain	0.004	0.005
France	0.002	0.002
Italy	0.001	0.002
Latvia	0.006	0.004
Lithuania	0.001	0.001
Luxemburg	0.001	0.001
Hungary	0.003	0.002
Netherlands	0.002	0.002
Austria	0.000	0.000
Poland	0.004	0.003
Portugal	0.000	0.001
Slovenia	0.000	0.001
Slovakia	0.005	0.005
Finland	0.000	0.000
Sweden	0.002	0.002
UK	0.003	0.005
EA	0.002	0.001
EU	0.002	0.002

Estimation period 2005.01–2021.12. SD denotes standard deviation

5 contains the average difference and the standard deviation of the difference between the two alternative ways of computing the geometric indicator of disagreement. It can be observed that the impact of including the share of ‘don’t know’ answers in the ‘no-change’ category when computing the disagreement indicator is almost imperceptible. The main reason for this finding lies in the fact that few responses fall within the *N* category in the consumer survey carried out by the European Commission.

The mean difference ranges from 0.000 to 0.006, with the only exception of Estonia where it reaches 0.01. Notwithstanding this, the evolution of the difference between both series tends to be always positive (with the exception of Denmark and Slovenia when for some periods the difference takes a negative value), but differs between countries. While in some countries it is high around the 2008 crisis, in other countries, the difference reaches the highest values from 2014 onwards.

## Appendix 2

Each month, the European Commission publishes seasonally adjusted balances of survey responses, but the series corresponding to each response category are only available in raw form, that is, the aggregate percentage of respondents in each category. Since both metrics of labour market uncertainty are computed from raw data, which are not seasonally adjusted, Claveria et al. (2021) proposed using a zero-phase low-pass filter to extract the periodicities of the survey responses that are closest to those observed in seasonally adjusted GDP. In this Appendix we used a Butterworth filter (Butterworth 1930) and replicated the whole empirical analysis for *D* and *N*. For clarity, we will denote both proxies of labour market uncertainty as *DB* and *NB*, as opposed to *DX* and *NX*, which were obtained using the X-13ARIMA-SEATS.

Figure 6 compares the evolution of each labour market uncertainty proxy for both filtering methods. Although both metrics covary during the sample period there are slight differences between them. Given that the Butterworth filter is a type of signal processing filter designed to have a frequency response as flat as possible in the pass band, the resulting series are less smooth that those obtained with X-13ARIMA-SEATS. See Proakis and Manolakis (1996) for detailed information on the properties of low-pass filtering.

Figure ^7^ shows the cross-correlograms between the two proxies of labour market uncertainty resulting from applying the Butterworth filter. As it could be expected, results do not differ much from those obtained with X-13ARIMA-SEATS (Fig. 2), albeit correlations obtained with the Butterworth filter tend to be slightly lower.

Finally, we repeated the NARDL analysis using Butterworth-filtered proxies of labour market uncertainty (*DB* and *NB*) and controlling for GDP year-on-year growth rates. We used the Chow and Lin (1971) interpolation technique to extract monthly data from quarterly GDP. Tables

**Table 6:** NARDL cointegration analysis results–Effect of *DB* on unemployment

Country	Type of asymmetry	Cointegration test *F* value	$\theta^{ + }$	$\theta^{ - }$
Austria	SR^HAC^	6.79**	− 0.0399	–
Belgium	none^HAC^	19.15**	− 0.2453**	–
Czechia	SR	11.06**	− 0.1158	–
Denmark	none	38.63**	− 0.0789	–
Estonia	SR,LR	16.62**	− 0.4078*	− 0.3334
Finland	SR^HAC^	15.59**	− 0.2405	–
France	SR^HAC^	8.07**	− 0.2706**	–
Germany	SR^HAC^	18.55**	− 0.0337	–
Greece	SR	14.21**	− 0.6638**	–
Hungary	none^HAC^	4.67	− 0.4553**	–
Italy	SR^HAC^	20.49**	− 0.6197**	–
Latvia	SR^HAC^	3.96	− 0.9140**	–
Lithuania	SR	9.32**	− 0.2740	–
Luxembourg	LR	1.70	0.0500	–
Netherlands	LR	11.15**	− 0.1348*	–
Poland	SR	3.04	− 0.1428	–
Portugal	SR^HAC^	6.52**	− 0.2283**	–
Slovakia	LR^HAC^	16.43**	− 0.1932**	− 0.1484**
Slovenia	none^HAC^	27.28**	− 0.2342**	–
Spain	none^HAC^	1.12	− 0.0943	–
Sweden	SR^HAC^	10.58**	− 0.4158**	–
UK	none^HAC^	2.60	− 0.0464	–
EA	SR^HAC^	2.12	− 0.0769	–
EU	SR	7.91**	− 0.2331**	–

**Significance at the 0.01 level, * at the 0.05 level. ^HAC^ denotes a model estimated using the Newey–West standard error correction due to autocorrelation and/or heteroscedasticity issues. ‘–’ denotes no negative effect, i.e. a unique (symmetric) long-run coefficient is estimated. Full set of results is available upon request

6 and

**Table 7:** NARDL cointegration analysis results–Effect of *NB* on unemployment

Country	Type of asymmetry	Cointegration test *F* value	$\theta^{ + }$	$\theta^{ - }$
Austria	SR^HAC^	8.60**	− 0.0466	–
Belgium	SR	8.27**	0.0268	–
Czechia	SR, LR	16.53**	− 0.4149**	− 0.2543*
Denmark	none^HAC^	18.44**	− 0.0996	–
Estonia	SR	21.31**	− 0.1440	–
Finland	SR^HAC^	18.93**	− 1.1351**	–
France	(SR, LR)^HAC^	4.20	− 0.5171**	− 0.5331**
Germany	SR^HAC^	13.30**	0.0104	–
Greece	SR	14.33**	− 0.2344	–
Hungary	none^HAC^	3.60	− 0.5383**	–
Italy	SR^HAC^	12.62**	0.0083	–
Latvia	(SR, LR)^HAC^	3.87	− 0.4906**	− 0.2982*
Lithuania	SR	9.01**	0.0694	–
Luxembourg	SR	3.06	0.1139	–
Netherlands	(SR, LR)^HAC^	6.68**	− 0.4313**	− 0.7477**
Poland	(SR, LR)^HAC^	7.73**	− 0.1099	− 0.0082
Portugal	none^HAC^	5.12	− 0.3147**	–
Slovakia	LR	13.56**	− 0.3475**	− 0.1342
Slovenia	LR^HAC^	9.95**	− 0.2266*	− 0.8970
Spain	none^HAC^	3.64	0.0175	–
Sweden	none^HAC^	13.84**	0.0200	–
UK	LR	6.45**	− 0.2588**	− 0.2836**
EA	SR^HAC^	4.80	0.1108**	–
EU	SR^HAC^	1.63	− 0.0111	–

**Significance at the 0.01 level, * at the 0.05 level. ^HAC^ denotes a model estimated using the Newey–West standard error correction due to autocorrelation and/or heteroscedasticity issues. ‘–’ denotes no negative effect, i.e. a unique (symmetric) long-run coefficient is estimated. Full set of results is available upon request

7 summarise the results for the impact of *DB* and *NB* on the unemployment rates of each country, and Figs. 8 and 9 show the results for the models in which we found a significant short- and/or long-run asymmetry according to the Wald test.

## Appendix 3

Finally, our measures of labour market uncertainty might possess valuable information for explaining other aspects of aggregate economic activity. To shed some light on this issue, we repeated the NARDL modelling approach presented through Eqs. (4), (5), (6), (7) and (8), using GDP year-on-year growth rates as the dependent variable, and each of our two uncertainty proxies (*DX* and *NX*) as regressors in separate specifications. The obtained results are presented in Tables

**Table 8:** NARDL cointegration analysis results–Effect of *DX* on GDP

Country	Type of asymmetry	Cointegration test *F* value	$\theta^{ + }$	$\theta^{ - }$
Austria	none^HAC^	2.86	0.3193	–
Belgium	SR^HAC^	3.80	− 0.3506*	–
Czechia	none^HAC^	2.85	− 0.0087	–
Denmark	none^HAC^	6.54*	0.5125**	–
Estonia	SR^HAC^	0.30	− 0.0979	–
Finland	none^HAC^	8.21**	0.7934**	–
France	none^HAC^	2.38	− 0.0731	–
Germany	none^HAC^	3.72	0.2511	–
Greece	none^HAC^	2.42	0.1767	–
Hungary	SR^HAC^	6.82*	0.7009**	–
Italy	SR^HAC^	11.82**	0.1765	–
Latvia	none^HAC^	5.96	0.8755**	–
Lithuania	none^HAC^	3.41	0.0782	–
Luxembourg	SR^HAC^	13.28**	− 0.1496	–
Netherlands	SR^HAC^	0.79	0.2342	–
Poland	SR^HAC^	5.38	0.2532	–
Portugal	none^HAC^	2.18	0.2261	–
Slovakia	(SR,LR)^HAC^	3.88	0.0404	0.1367
Slovenia	none^HAC^	4.98	0.0027	–
Spain	SR^HAC^	2.56	0.3493	–
Sweden	none^HAC^	10.50**	0.5415**	–
UK	SR^HAC^	3.43	− 0.0816	–
EA	none^HAC^	2.96	0.4247	–
EU	SR^HAC^	3.62	0.7893	–

**Significance at the 0.01 level, * at the 0.05 level. ^HAC^ denotes a model estimated using the Newey–West standard error correction due to autocorrelation and/or heteroscedasticity issues. ‘–’ denotes no negative effect, i.e. a unique (symmetric) long-run coefficient is estimated. Full set of results is available upon request

8 and

**Table 9:** NARDL cointegration analysis results–Effect of *NX* on GDP

Country	Type of asymmetry	Cointegration test *F* value	$\theta^{ + }$	$\theta^{ - }$
Austria	SR^HAC^	3.69	− 0.4028*	–
Belgium	SR^HAC^	6.75*	− 0.0987	–
Czechia	none^HAC^	2.48	0.0281	–
Denmark	SR^HAC^	2.11	− 0.0571	–
Estonia	none^HAC^	3.36	− 0.2341	–
Finland	SR^HAC^	9.59**	0.0096	–
France	SR^HAC^	3.99	− 0.2464	–
Germany	SR^HAC^	8.04**	0.0548	–
Greece	SR^HAC^	2.13	− 0.0987	–
Hungary	none^HAC^	5.99	1.2478**	–
Italy	none^HAC^	6.51*	0.0121	–
Latvia	SR^HAC^	3.49	− 0.0614	–
Lithuania	none^HAC^	6.56*	− 0.2017	–
Luxembourg	SR^HAC^	7.22*	− 0.3826	–
Netherlands	none^HAC^	5.10	− 0.0942	–
Poland	(SR, LR)^HAC^	3.32	0.6580*	0.7492*
Portugal	SR^HAC^	2.69	0.2462	–
Slovakia	none^HAC^	3.96	− 0.2251	–
Slovenia	SR^HAC^	9.61**	0.3505	–
Spain	none^HAC^	3.23	0.2304	–
Sweden	none^HAC^	5.76	− 0.02244	–
UK	SR^HAC^	2.37	− 0.1056	–
EA	SR^HAC^	4.65	0.0638	–
EU	none^HAC^	5.91	− 0.0742	–

**Significance at the 0.01 level, * at the 0.05 level. ^HAC^ denotes a model estimated using the Newey–West standard error correction due to autocorrelation and/or heteroscedasticity issues. ‘–’ denotes no negative effect, i.e. a unique (symmetric) long-run coefficient is estimated. Full set of results is available upon request

9, and Figs. 10 and 11.

The analysis reveals a significant long-run relationship far more seldom than in the models with unemployment as the target variable. This comes as no surprise, since both uncertainty proxies were specifically based on consumers’ assessments of their unemployment expectations, not the general economic outlook in the country. Nevertheless, for countries with a significant relationship, uncertainty parameters are largely positive (see Tables 8 and 9). Once again, this corroborates the cyclical nature of our uncertainty proxies. Consumers tend to produce the most homogeneous unemployment assessments specifically during economic crises (compare to Fig. 2).

## Appendix 4

To further assess the robustness of our results, we estimate a VAR model of the Bloom (2009) type with a recursive identification scheme. Although the dynamic multipliers presented in Figs. 10 and 11 are not directly comparable to impulse response functions (IRFs) in vector autoregression (VAR) models, it is easily observable that the asymmetries—the differences between positive and negative effects of uncertainty on GDP—highly resemble the well-known J-curve shape of the responses of economic activity to a unit shock in economic uncertainty (see e.g. Bloom 2009). The only difference is that, this time, *DX* and *NX* mostly exhibit positive effects on GDP, which diminish shortly after the initial shock, and then fade away to zero. The evidence presented by Bloom (2009) or Baker et al. (2016) for other similar uncertainty indicators such as the Economic Policy Uncertainty (EPU) Index or different financial volatility indicators is directly comparable, with the difference that their effects are of the opposite sign (i.e. negative).

As the final robustness check, we applied a VAR specification for the EA, adopted from Bloom (2009), with a recursive identification scheme. We estimated several versions of the model, questioning the robustness of our results. In model 1, the following Cholesky ordering was used: log(Euro Stoxx 50 stock market index), *D* as an uncertainty proxy, 3-month money market interest rate, log(labour cost index; wages and salaries), log(Harmonized Index of Consumer Prices), log(employment, in thousands), log(GDP). In model 2, we used the unemployment rate (UN) instead of employment. Model 3 differs from model 1 by using *N* as an uncertainty proxy, while model 4 differs from model 2 in the same manner. All models are estimated for the time period 2007.04–2021.12, conditioned by the availability of the Euro Stoxx 50 index. All variables are seasonally adjusted using the ARIMA-X13 method. The Euro Stoxx 50 index is obtained from Yahoo Finance, while all other variables are obtained from Eurostat.

Directly following Bloom (2009), all variables are Hodrick–Prescott filtered (expressed in deviations from the long-term trend, with a smoothing parameter equal to 129,600). The main results—IRFs of labour costs, UN, and GDP to a one-standard deviation increase in uncertainty—are presented in Figs. 12, 13, 14. Once again, these results largely corroborate our previous findings. Figure 12 reveals that labour costs also exhibit a common J-curve effect in response to an uncertainty shock. Following a shock to both uncertainty proxies, UN decreases rapidly (Fig. 12) and GDP promptly increases (Fig. 13). Then, the effect of uncertainty diminishes and slowly decays. The only exception to this finding is model 3 in Fig. 13, where the impact of *N* on UN moves to positive territory shortly after the initial shock.

## Declarations

### Human and animals rights

This research did not involve human participants and/or animals.
